# Overlapping functions and protein-protein interactions of LRR-extensins in Arabidopsis

**DOI:** 10.1371/journal.pgen.1008847

**Published:** 2020-06-19

**Authors:** Aline Herger, Shibu Gupta, Gabor Kadler, Christina Maria Franck, Aurélien Boisson-Dernier, Christoph Ringli

**Affiliations:** 1 Institute of Plant and Microbial Biology, University of Zurich, Zurich, Switzerland; 2 Biocenter, Botanical Institute, University of Cologne, Cologne, Germany; Wake Forest University, UNITED STATES

## Abstract

Plant cell growth requires the coordinated expansion of the protoplast and the cell wall, which is controlled by an elaborate system of cell wall integrity (CWI) sensors linking the different cellular compartments. LRR-eXtensins (LRXs) are cell wall-attached extracellular regulators of cell wall formation and high-affinity binding sites for RALF (Rapid ALkalinization Factor) peptide hormones that trigger diverse physiological processes related to cell growth. LRXs function in CWI sensing and in the case of LRX4 of *Arabidopsis thaliana*, this activity was shown to involve interaction with the transmembrane *C**atharanthus* *r**oseus* Receptor-Like Kinase1-Like (CrRLK1L) protein FERONIA (FER). Here, we demonstrate that binding of RALF1 and FER is common to most tested LRXs of vegetative tissue, including LRX1, the main LRX protein of root hairs. Consequently, an *lrx1*-*lrx5* quintuple mutant line develops shoot and root phenotypes reminiscent of the *fer-4* knock-out mutant. The previously observed membrane-association of LRXs, however, is FER-independent, suggesting that LRXs bind not only FER but also other membrane-localized proteins to establish a physical link between intra- and extracellular compartments. Despite evolutionary diversification of various LRX proteins, overexpression of several chimeric *LRX* constructs causes cross-complementation of *lrx* mutants, indicative of comparable functions among members of this protein family. Suppressors of the pollen-growth defects induced by mutations in the CrRLK1Ls *ANXUR1/2* also alleviate *lrx1 lrx2*-induced mutant root hair phenotypes. This suggests functional similarity of LRX-CrRLK1L signaling processes in very different cell types and indicates that LRX proteins are components of conserved processes regulating cell growth.

## Author summary

Cell growth in plants requires the coordinated enlargement of the cell and the surrounding cell wall, which is regulated by an elaborate system of cell wall integrity sensors, proteins involved in the exchange of information between the cell and the cell wall. In *Arabidopsis thaliana*, LRR-extensins (LRXs) are localized in the cell wall and bind RALF peptides, hormones that regulate cell growth-related processes. LRX4 also binds the plasma membrane-localized protein FERONIA (FER), thereby establishing a link between the cell and the cell wall. Here, we show that membrane association of LRX4 is not dependent on FER, suggesting that LRX4 binds other, so far unknown proteins. The LRR domain of several LRXs can bind to FER, consistent with the observation that mutations in multiple *LRX* genes are required to recapitulate a *fer* knock-out phenotype. Our results support the notion that LRX-FER interactions are key to proper cell growth.

## Introduction

The plant cell wall is a complex network of interwoven polysaccharides and structural proteins that supports plant structure and protects the cell from biotic and abiotic stresses [1]. Importantly, it serves as a shape-determining structure that resists the internal turgor pressure emanating from the vacuole. Cell growth requires a tightly regulated expansion of the cell wall, generally accompanied by the concomitant biosynthesis of new cell wall material that is integrated into the expanding cell wall [2]. The signal transduction machinery required for coordinating the intra- and extracellular processes involves a number of transmembrane proteins at the plasma membrane to connect the different cellular compartments [3]. Among these, the *C**atharanthus* *r**oseus* Receptor-Like Kinase 1-Like protein (CrRLK1L) THESEUS1 (THE1) acts as a Cell Wall Integrity (CWI) sensor that perceives reduced cellulose content in the cell wall and induces compensatory changes in cell wall composition to restrain growth [4]. Several members of the CrRLK1L family are involved in cell growth processes [5,6,7,8,9]. FERONIA (FER) is required for successful pollen tube reception during the fertilization process involving local disintegration of the cell wall to release sperm cells [10]. The extracellular domain of FER has been demonstrated to bind pectin, a major component of cell walls [11]. This interaction could contribute to the function of FER during CWI sensing and perception of mechanical stresses [11,12,13]. Several CrRLK1L receptors have been demonstrated to bind Rapid ALkalinization Factor (RALF) peptides that are involved in the alkalinization of the extracellular matrix, the change of Ca^2+^ fluxes and the modulation of cell growth and response to pathogens [14,15,16,17,18,19,20,21]. Hence, CrRLK1L proteins appear to have multiple functions, suggesting that their activity is at the nexus of different cellular processes.

RALF1 was identified as a ligand of FER and a number of additional proteins are involved in the RALF1-FER triggered signaling process, either as signaling components such as ROP2, ROPGEF, ABI2, RIPK [22,23,24,25], as co-receptors such as BAK1 and LLG1/2 [22,26,27], or as targets of the FER-dependent pathway, such as AHA2 [15]. The receptor-like cytoplasmic kinase MARIS (MRI) and the phosphatase ATUNIS1 (AUN1) were identified as downstream components of signaling activities induced by the pollen-expressed ANXUR1 (ANX1) and ANX2, the closest homologs of FER [6,28,29]. Both AUN1 and MRI also influence root hair growth, indicating that they function downstream of a CrRLK1L protein in root hairs and MRI has been demonstrated to function downstream of FER [28]. Apart from this observation, it remains unclear to what extent the signaling components are shared among the different CrRLK1Ls.

**Fig 1:**
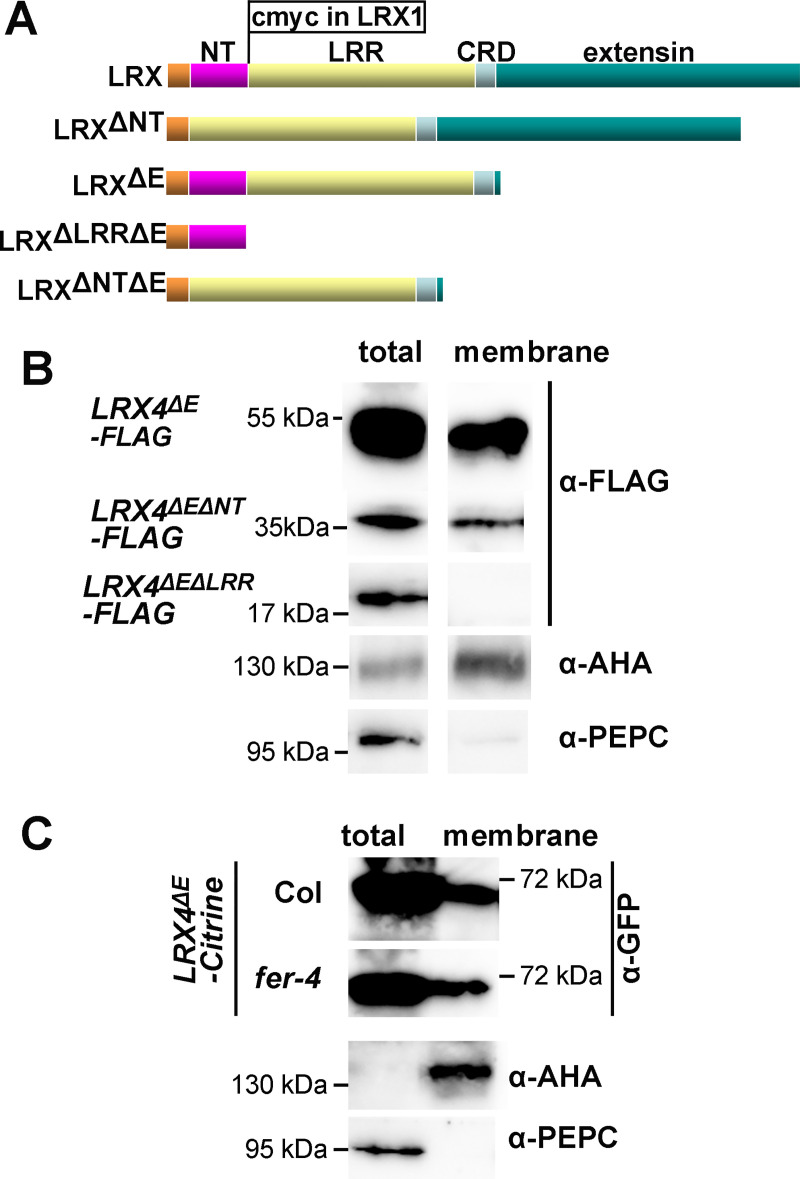
Membrane association of variants of LRX proteins. (A) LRX proteins consist of a signal peptide for export of the protein (light brown), an NT-terminal domain (purple) of unknown function, an leucine-rich repeat (LRR) domain (yellow), a Cys-rich hinge region (CRD), and a C-terminal extensin domain (green), with Ser-Hypn repeats typical of hydroxyproline-rich glycoproteins, for insolubilization of the protein in the cell wall. The different deletion constructs used in this study are listed, with”Δ” indicating deleted domains. In the LRX1 construct, a cMyc tag was introduced between the NT- and the LRR-domain, which does not interfere with protein function and allows for immuno-detection of LRX1. (B and C) Immunoblots of total extracts (left) and membrane fractions (right) of transgenic Arabidopsis expressing *LRX4* constructs as indicated. AHAs are transmembrane proteins used as plasma membrane marker proteins. PEPC is the cytosolic PEP carboxylase used as a cytosolic marker.

LRXs (LRR-eXtensins) are extracellular proteins involved in cell wall formation and cell growth. They consist of an N-terminal (NT) domain and a Leucine-rich repeat (LRR) of 11 repeats, followed by a short Cys-rich domain (CRD) that serves as a linker to the C-terminal extensin domain (Fig 1A) [30]. The extensin domain contains Ser-Hyp_n_ repetitive sequences that are characteristic for hydroxyproline-rich glycoproteins [31,32] and appears to serve in anchoring the protein in the extracellular matrix [33,34]. The *LRX* family of Arabidopsis consists of eleven members, which can be grouped according to three specific expression patterns. *LRX1/2* are predominantly expressed in root hairs, *LRX3/4/5* in the main root and the shoot, and *LRX8/9/10/11* in pollen. Mutations in these genes cause cell wall perturbation and cell growth defects in the respective cell types [33,35,36,37,38,39]. *lrx1* mutants develop deformed root hairs that are swollen, branched, and frequently burst [33]. This phenotype is strongly aggravated in the *lrx1 lrx2* double mutant that is virtually root hair-less [35]. The *lrx345* triple mutant shows defects in vacuole development, monitoring of cell wall modifications, and sensitivity to salt stress that are reminiscent of the *fer-4* knock-out mutant, and LRX4 physically interacts with FER [40,41]. Therefore, these results suggest that LRXs and FER function in a common process. The N-terminal moiety with the NT- and LRR-domains has been shown to associate with the membrane fraction [37], indicating a function of LRXs in linking the cell wall with the plasma membrane by binding of a membrane-localized interaction partner.

LRX proteins were recently identified as high-affinity binding sites for RALF peptides, with the binding spectrum differing among the LRXs. LRX8 of pollen tubes was shown to physically interact with RALF4 [42], while in vegetative tissues, the root/shoot-expressed LRX3, LRX4, and LRX5 were reported to bind RALF1,22,23,24, and 31 [40,41]. Whether and how the binding of RALFs and FER to LRXs influences the interaction of these proteins remains to be investigated. It is also unknown to what extent the different LRXs are functionally similar and whether they share FER as a common interaction partner.

Here, we demonstrate that the root hair-expressed LRX1 binds FER and RALF1, like most of the LRX proteins that function in vegetative tissues. Together with the *fer*-like phenotype of higher-order *lrx* mutants, this suggests that the LRX-FER module is conserved in vegetative tissues. Cross-complementation experiments of *lrx* mutants with overexpression constructs encoding chimeric proteins with the NT-LRR domains of different LRXs fused to the extensin domain of LRX1 indicate that most but not all LRX NT-LRR domains exert similar functions. The membrane association of LRXs, however, is independent of binding to FER, suggesting that additional binding activities of LRX proteins are relevant for their activity in the regulation of cell growth and CWI maintenance.

## Results

### The membrane association of LRX4 is independent of FER

We have previously shown that apoplastic LRX proteins associate with the plasma membrane and LRX4 binds to the transmembrane CrRLK1L FER [37,41]. This property of LRXs was investigated in more detail. *LRX* constructs lacking the extensin coding sequence (*LRX4*^ΔE^) were used in this and in later experiments to prevent insolubilization of the LRX protein in the cell wall. To determine the protein domain(s) necessary for membrane-association, *LRX4^ΔE^-FLAG* (coding for LRX4 missing the extensin domain), *LRX4^ΔLRRΔE^-FLAG* (coding for LRX4 missing the LRR- and the extensin domain), and *LRX4^ΔNTΔE^-FLAG* (coding for LRX4 missing the NT- and the extensin domain) (Fig 1A) were expressed under the *CaMV35S* (*35S*) promoter in transgenic Arabidopsis. The calculated molecular weight of all recombinant proteins are shown in S1 Table. Membrane fractions of the different transgenic lines were prepared from 10 days-old seedlings and tested for presence of the recombinant proteins. As controls, antibodies against plasma membrane-localized (α-AHA) and cytoplasmic (α-PEPC) proteins were used to confirm successful enrichment of the membrane fraction. As shown in Fig 1B, all proteins were present in the total fraction. LRX4^ΔE^-FLAG and LRX4^ΔNTΔE^-FLAG were also detected in the membrane fraction, but not LRX4^ΔLRRΔE^-FLAG. Hence, membrane association of LRX4 depends on the presence of its LRR domain that also binds FER [41], which raises the question whether membrane association of LRX4 depends on its interaction with the plasma membrane-localized FER. We investigated this using a wild type and *fer-4* mutant line producing an LRX4^ΔE^-Citrine fusion protein. As for LRX4^ΔE^-FLAG, also LRX4^ΔE^-Citrine was detected in the membrane fraction. This localization, however, was not dependent on FER, as it was also observed in the *fer-4* mutant background (Fig 1C). Since the LRR domain is unlikely to directly interact with membranes, membrane association of LRX4 appears to involve other proteins.

### Multiple LRXs of vegetative tissue interact with FER

**Fig 2:**
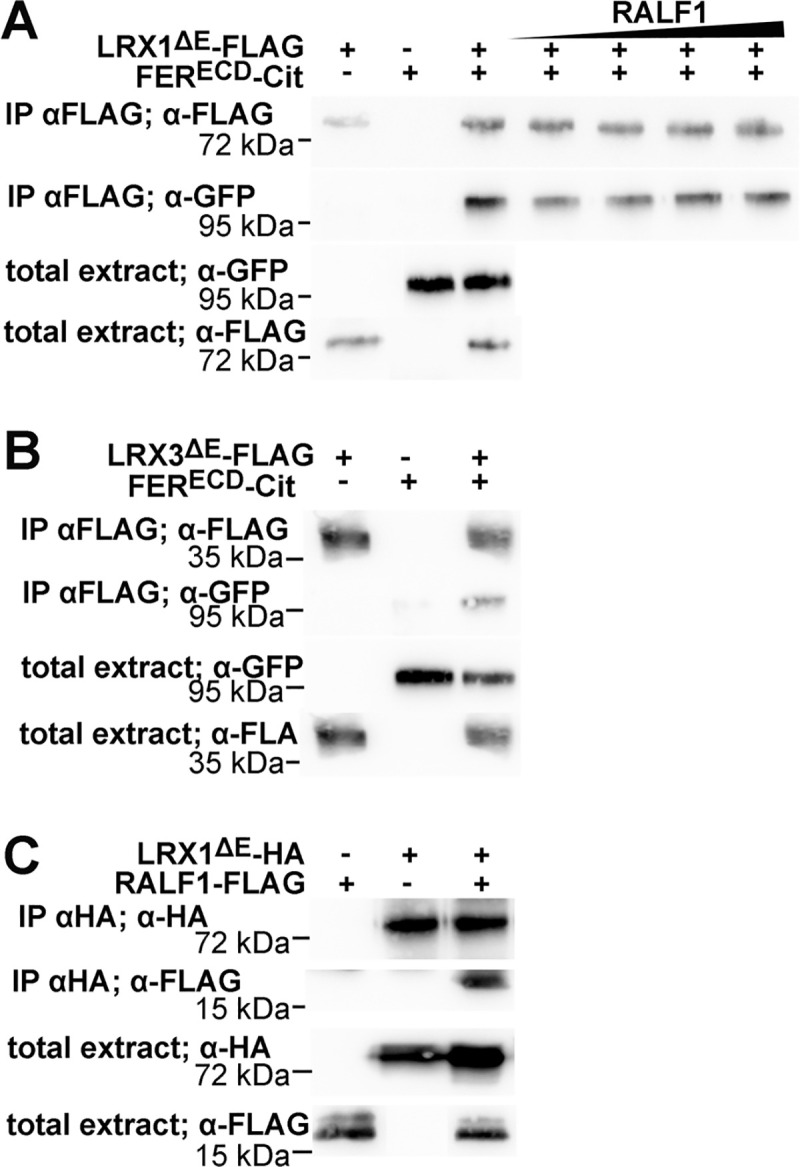
LRX interaction with FER and RALF1. Immunoblots of Co-IP experiments with proteins expressed in *N*. *benthamiana*. (A and B) Both LRX1^ΔE^ and LRX3^ΔE^ show interaction with FER^ECD^. Addition of synthetic RALF1 peptide (50, 100, 200, 1000 nM) to extracts does not influence the interaction of LRX1^ΔE^ with FER^ECD^. (C) LRX1^ΔE^ shows binding to RALF1. Antibodies used for IP and subsequent detection by immunoblotting are indicated. RALF1-FLAG and FER^ECD^-Citrine run slower than according to their molecular weight of 9.5 and 75.7 kDa, respectively, whereas LRX1^ΔE^ runs higher than expected (54 kDa).

The root hair-localized LRX1 is so far the best characterized LRX protein and the *lrx1* root hair mutant represents a convenient genetic system for analyses of LRX protein function [33,34,43,44]. It was previously reported that LRX4 and FER interact [41], but since FER was reported to be necessary for CWI maintenance in growing root hairs [23,28], it was of interest to test whether LRX1 also interacts with FER. To this end, constructs encoding LRX1^ΔE^-FLAG and the extracellular domain of FER fused to citrine (FER^ECD^-Citrine) under the *35S* promoter were expressed in tobacco for Co-IP experiments. LRX1^ΔE^ showed interaction with FER^ECD^ (Fig 2A), which was confirmed in a yeast-two-hybrid (Y2H) experiment (S1 Fig). The Y2H experiments were extended to other LRXs of vegetative tissues, namely the LRR domains of LRX2, LRX3, LRX4, and LRX5. LRX2, LRX4, and LRX5 showed interaction with FER^ECD^ (S1 Fig), confirming the previous finding of LRX4 interacting with FER^ECD^ [41]. Due to technical problems with LRX3 LRR expression in the Y2H system, we conducted Co-IP experiments with LRX3^ΔE^ and FER^ECD^, which revealed interaction of the two proteins (Fig 2B). Thus, the LRR domains of all LRXs of vegetative tissues that were tested so far show interaction with FER^ECD^.

### RALF1 binds with high affinity to LRX1, LRX4, and LRX5

LRX4 has been shown to bind RALF1, a peptide hormone that also interacts with FER [15,41]. Here, we tested binding of RALF1 to LRX1. Transient expression of *LRX1^ΔE^-HA* and *RALF1-FLAG* in *N*. *benthamiana* followed by Co-IP and immunoblotting showed interaction of the two proteins (Fig 2C). This was confirmed by Y2H, where under selective conditions, yeast cells grew effectively in the presence of the two proteins (S2 Fig).

The kinetics of the interaction of LRX proteins with RALF1 were tested with Biolayer Interferometry (BLITZ). The LRX^ΔE^-FLAG proteins of LRX1, LRX3, LRX4, and LRX5 used for this experiment were expressed transiently in tobacco. Accumulation of all proteins to comparable levels was confirmed by immunoblotting prior to BLITZ analysis. For RALF1, *in vitro* synthesized peptide was used. Biological activity of the peptide was tested and confirmed in root growth assays where application of RALF1 effectively inhibited root growth of Arabidopsis seedlings (S3 Fig). The BLITZ analysis revealed a dissociation constant Kd of around 5 nM for the interaction of LRX1, LRX4, and LRX5 with RALF1 (S4 Fig), whereas LRX3 showed a much lower affinity (Table 1). While the LRX^ΔE^-FLAG had to be soluble in order to get purified prior to application on the sensor (for details, see Materials and Methods), it cannot be completely ruled out that LRX3^ΔE^-FLAG has a reduced solubility in this experiment that would provide an alternative explanation for the observed high Kd.

**Table 1:** Dissociation constant of LRXs-RALF1 interactions.

LRX protein	RALF1 Kd (nM)
LRX1	4.5
LRX3	11’000
LRX4	5.5
LRX5	3.5

Kd was determined by BLITZ, using purified *LRX^ΔE^* expressed in *N*. *benthamiana*.

In a next step, the dynamics of LRX-RALF1-FER interaction was investigated with a focus on LRX1. To this end, extracts used to show Co-IP of LRX1^ΔE^ and FER^ECD^ were supplemented with increasing concentrations of RALF1 peptide. The addition of RALF1, however, did not affect the interaction between LRX1^ΔE^ and FER^ECD^ (Fig 2A), suggesting that RALF1 does not affect LRX1-FER interaction.

### The *lrx12345* quintuple mutant mimics the *fer-4* mutant phenotype

**Fig 3:**
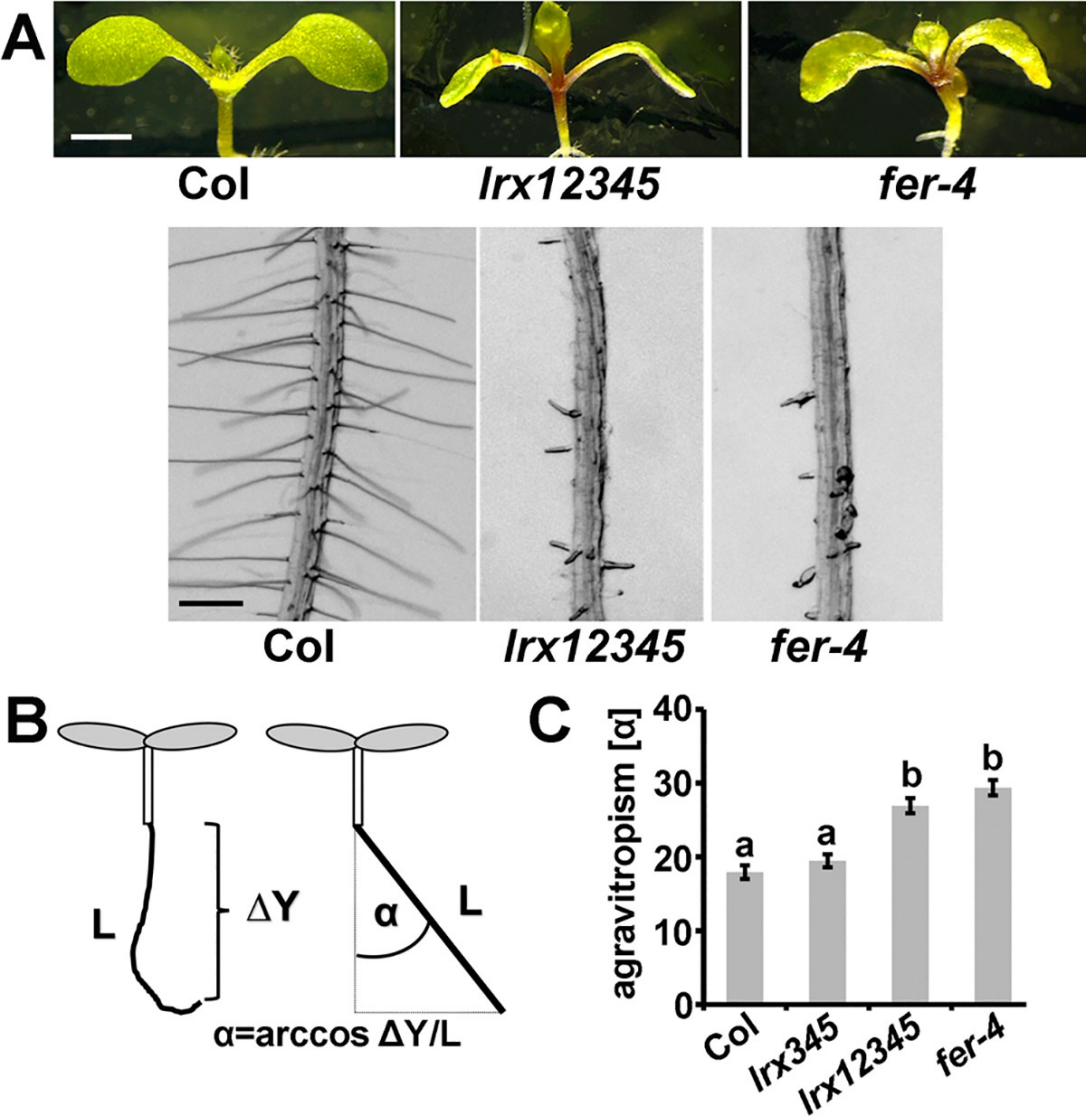
*fer-4* and *lrx12345* mutants show comparable phenotypes. (A) Seedlings were grown for 5 days on half-strength MS for analysis of root hair formation (bottom) and another 5 days for analysis of shoot development (top). (B) Quantification of gravitropic response by the root growth index. (C) Increasing agravitropy by accumulation of *lrx* mutations, represented by the angle α as shown in (B). Error bars represent SEM. Different letters above bars indicate significant differences (T-test, n>20, P<0.0001). Bar = 3 mm (A top); 0.5 mm (A bottom)

Mutations in FER such as *fer-4* exhibit both root and shoot phenotypes [23,40,41]. The results described above suggest that the five LRX proteins produced in vegetative tissues exert overlapping functions. Since a double mutant for the root hair-expressed *LRX1* and *LRX2* displays a root hair phenotype comparable to the knock-out mutant *fer-4* [23,35], and the *lrx345* triple mutant develops a shoot phenotype that is reminiscent of *fer-4* [40,41], we anticipated that an *lrx12345* quintuple mutant would be globally similar to *fer-4*. The *lrx1 lrx2* mutant was crossed with the *lrx345* triple mutant and an *lrx12345* quintuple mutant was identified in the segregating F_2_ population of this cross based on a root hair-less phenotype and delayed shoot growth with an increase in anthocyanin content. Indeed, the *lrx12345* quintuple mutant shows *fer-4* like phenotypes in the root and shoot at the seedling stage and increased accumulation of anthocyanin compared to the wild type (Fig 3A). *fer-4* seedlings grown in a vertical orientation display reduced gravitropic growth of the root [45]. This growth defect was assessed in the wild type, *fer-4*, and different *lrx* mutant combinations by assessing the vertical growth index [46]. For quantification, the ratio between the absolute root length and the progression of the root along the gravity vector, the arccos of α, was used as illustrated in Fig 3B. The quintuple *lrx12345* mutant develops an agravitropic response comparable to *fer-4* (Fig 3C). Altogether, our results indicate that the LRXs of vegetative tissues interact with and are relevant for several activities of FER.

### The LRR- and NT-domains are necessary for LRX1 function

**Fig 4:**
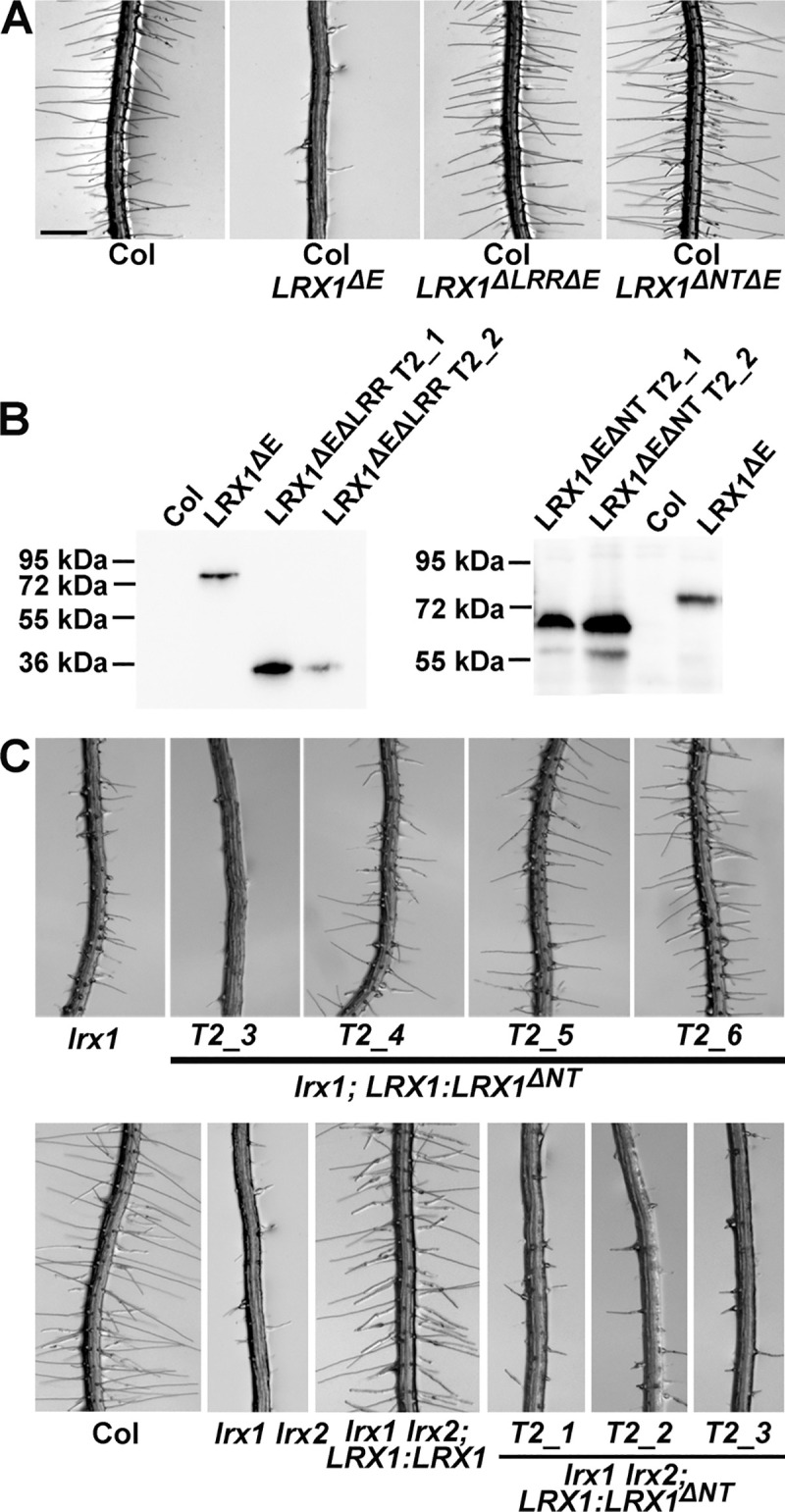
Both the LRR and NT domains are required for function of LRX1. (A) Root-hair phenotype of wild type seedlings (Col) or transgenic Col lines expressing *LRX1^ΔE^*, *LRX1^ΔLRRΔE^*, or *LRX1^ΔNTΔE^* (for protein structures, see Fig 1A). The dominant-negative effect induced by *LRX1^ΔE^* depends on both the LRR and the NT domains. A representative example of several independent transgenic lines is shown. (B) Immunoblots using the anti-cMyc antibody 9E10 detecting the proteins encoded by the transgenic lines shown in (A). (C) In the absence of the NT domain (*LRX1^ΔNT^*) complementation of *lrx1* gives variable phenotypes (upper lane) and no complementation of *lrx1 lrx2* is observed. An immunoblot showing LRX1^ΔNT^ accumulation in the different lines is shown in S5 Fig. Bar = 0.5 mm (A, C).

Expression of a truncated LRX1 version lacking the extensin coding sequence (LRX1^ΔE^; Fig 1A) under the *LRX1* promoter induces a dominant-negative effect in wild-type seedlings, resulting in a defect in root hair formation [34], possibly because LRX1^ΔE^ competes with the endogenous LRX1 for binding partners. This observed activity of LRX1^ΔE^ was used for further functional analysis of the LRX1 protein. Specifically, we assessed which domains are required or dispensable for the dominant-negative effect. The LRR- or the NT-domain were removed from the LRX1^ΔE^ construct, resulting in LRX1^ΔLRRΔE^ and LRX1^ΔNTΔE^, respectively (Fig 1A). The corresponding constructs under control of the *LRX1* promoter were transformed into wild-type plants. T_2_ seedlings expressing either of the two constructs developed wild-type root hairs (Fig 4A), hence failed to produce the dominant-negative effect on root hair formation. Extracts from root tissue of the different lines were used for immunoblotting. An antibody detecting the cMyc-tag of the recombinant LRX1 variants confirmed that the proteins were produced. As shown in Fig 4B, the transgenic lines yield proteins with the expected decrease in mass of LRX1^ΔE^ > LRX1^*ΔNT*ΔE^ > LRX1^*ΔLRR*ΔE^. Taken together, this indicates that both the LRR- and the NT-domains are required but neither is sufficient to induce the dominant-negative effect on root hair development.

In a complementary approach, we tested whether the NT-domain is required for the function of the full-length LRX1. To this end, the *lrx1* and *lrx1 lrx2* mutants developing intermediate and strong root hair defects, respectively [35], were transformed with the constructs *LRX1*:*LRX1* and *LRX1*:*LRX1^ΔNT^*. Unlike the full-length *LRX1* which complements the *lrx1* mutant [33,34], complementation with *LRX1*:*LRX1^ΔNT^* produced inconsistent results. A number of independent T_2_ families were analyzed, some showing complementation of *lrx1*, i.e. wild type-like root hair formation, whereas others developed a stronger defect comparable to the *lrx1 lrx2* double mutant (Fig 4C). Detection of *LRX1^ΔNT^* by immunoblotting revealed no correlation between protein abundance and root hair formation (S5 Fig). In *lrx1 lrx2* mutants, the full-length *LRX1*:*LRX1* induced wild type-like root hairs whereas *LRX1*:*LRX1^ΔNT^* consistently failed to reestablish root hair development (Fig 4C) despite accumulation of the transgene-encoded protein (S5 Fig). Hence, the absence of the NT-domain does not completely abolish protein activity but interferes with the function of LRX1.

### Functional equivalence among different LRX proteins

**Fig 5:**
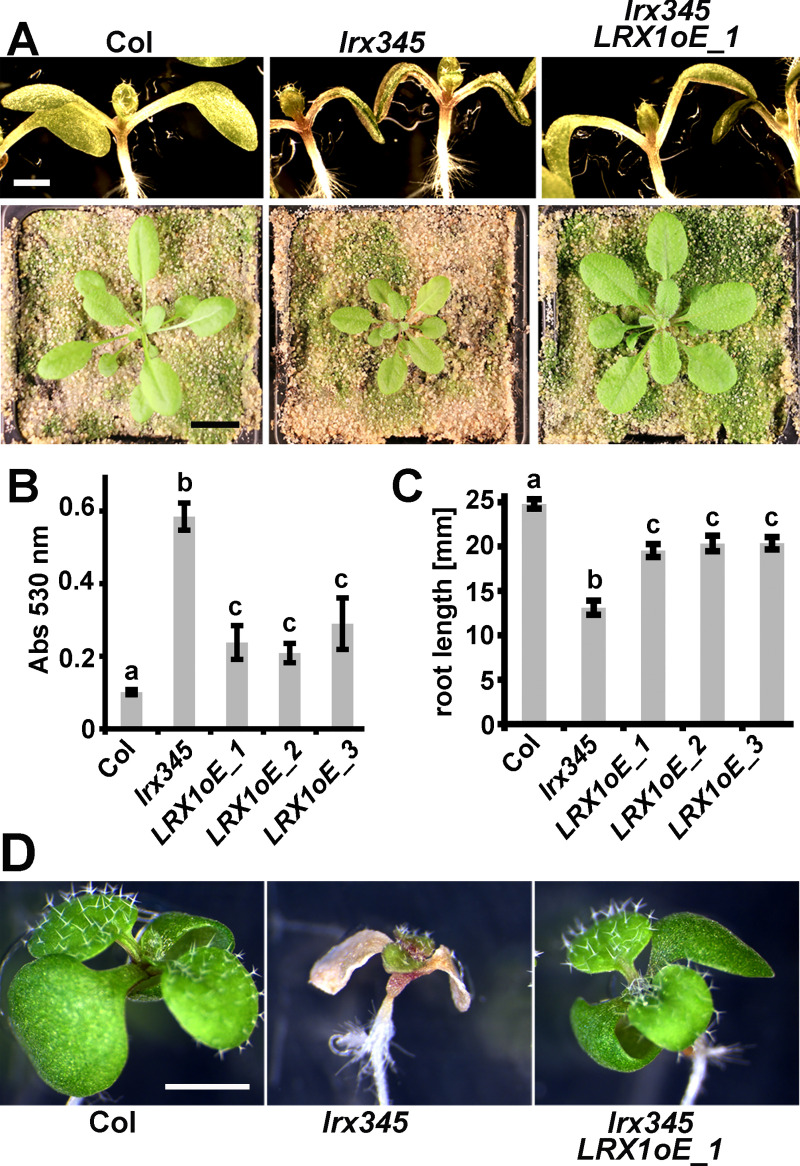
Functional redundancy among LRX proteins. (A-C) Complementation of the *lrx345* triple mutant with the *35S*:*LRX1* (*LRX1oE*) construct. Representative examples are shown. (A) Seedling shoots after 7 days of growth on half-strength MS (upper lane) and plants grown in soil (lower lane). (B) Anthocyanin accumulation in 12-days-old seedlings is increased in the *lrx345*. (C and D) The *lrx345* mutant seedlings grown in the presence of 100 mM NaCl show increased salt sensitivity, resulting in shorter roots (C) and impairment of shoot growth (D) compared to control Col. These phenotypes are alleviated by *LRX1* overexpression. Error bars represent SEM; different letters above the graphs indicate significant differences (T-test, N>20, P<0.05). Bars = 3 mm (A top); 1 cm (A bottom), 2 mm (D).

Analyses performed so far suggested that most vegetatively-expressed LRX proteins have comparable functions with the possible exception of LRX3 that appears to poorly bind RALF1. To compare *in planta* the function and activity of *LRX* genes expressed in different tissues, trans-complementation experiments were performed. To this end, the genomic coding sequence of *LRX1* including the cMyc-tag (Fig 1A) was cloned into an overexpression cassette containing the *35S* promoter and the resulting *35S*:*LRX1* construct was used for transformation of the *lrx345* triple mutant. Several independent homozygous transgenic lines were identified and characterized. Semi-quantitative RT-PCR confirmed expression of the transgene in the lines (S6A Fig). For assessment of the complementation of the *lrx345* phenotype, alterations in plant growth and physiology were used as parameters. *lrx345* mutants are smaller than the wild type both at the seedling stage and at later stages when grown in soil [36]. This phenotype is alleviated in the *35S*:*LRX1* transgenic lines (Fig 5A, S7A Fig). The increased anthocyanin accumulation in *lrx345* mutant seedlings compared to the wild type is significantly reduced in transgenic lines (Fig 5B), and the salt-hypersensitivity of shoot and root growth in the *lrx345* triple mutant [40] was also alleviated in the transgenic lines (Fig 5C and 5D). Hence, the *35S*:*LRX1* construct can largely rescue the *lrx345* mutant phenotypes.

**Fig 6:**
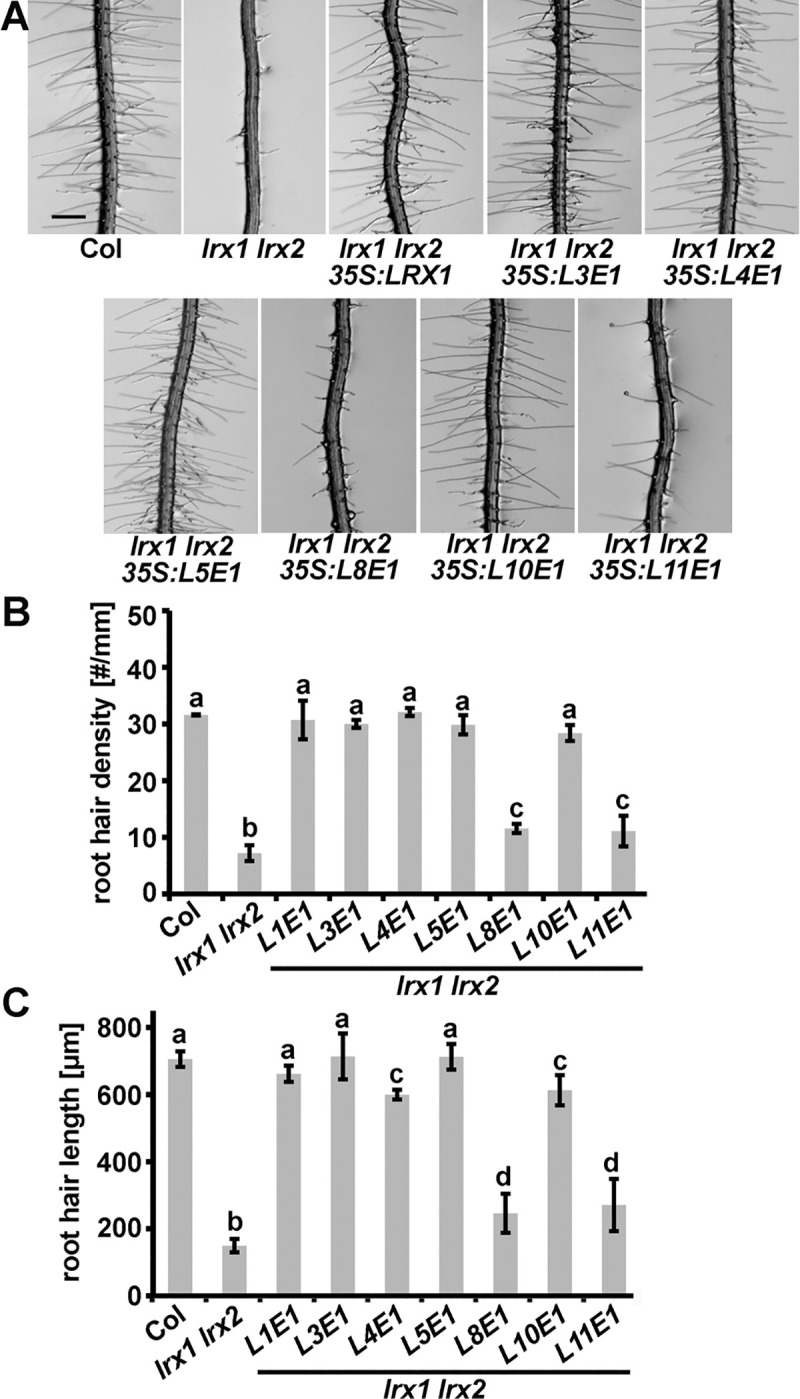
Complementation of the *lrx1 lrx2* phenotype by different *LRXs*. (A) Complementation of the *lrx1 lrx2* root hair defect by chimeric construct of *L3*,*4*,*5*,*8*,*10*, and *11* (ATG start codon to CRD) fused to the extensin coding sequence of *LRX1* (*E1*) under the *35S* promoter. Representative examples of several independent transgenic lines for each construct are shown. Bar = 0.5 mm. Quantification of the complementation by assessing root hair density (B) and root hair length (C) in the different lines. Seedlings were grown for 5 days on half-strength MS in a vertical orientation. Error bars represent SEM; different letters above the graphs indicate significant differences (T-test, N = 50, P<0.01).

In a complementary experiment, rescue of the intermediate root hair phenotype of the *lrx1* mutant, and the strong root hair phenotype of the *lrx1 lrx2* mutant [35] with overexpression of *LRX3*, *LRX4*, and *LRX5* was tested. The extensin coding sequences of *LRX3*, *LRX4*, and *LRX5* could not be stably maintained in *E*.*coli*. Therefore, and as previously described [36], the extensin-coding domains of the *LRX3*,*4*,*5* genes were replaced by that of *LRX1*. The resulting chimeric genes are referred to as *L3E1*, *L4E1*, and *L5E1* (L and E referring to the N-terminal moiety from the start codon to the CRD (Fig 1A) and the extensin coding sequence, respectively). The constructs encoding the chimeric proteins were placed under the control of the *35S* promoter and were transformed into the *lrx1* and *lrx1 lrx2* mutants. For each of the three constructs, several independent T_2_ lines were identified, all of which showed expression of the transgene at the transcriptional level (S6B Fig). The root hair growth defect of the *lrx1* mutant (S7B Fig) as well as the stronger *lrx1 lrx2* double mutant phenotype (Fig 6A) were suppressed by all of the three chimeric constructs.

The pollen-expressed *LRX8-LRX11* [37] form a separate phylogenetic clade and are more similar to pollen-expressed *LRXs* of other plant species than to vegetative *LRXs* of Arabidopsis [47,48]. It was tested whether the N-terminal moieties of these LRXs in combination with the extensin domain of LRX1 are functionally comparable to LRX1. The chimeric constructs *L8E1*, *L10E1*, and *L11E1* under the *35S* promoter were transformed into the *lrx1* single and *lrx1 lrx2* double mutant. Several independent transgenic T_2_ lines were produced for each construct and transgene expression was confirmed in the lines (S6C Fig). Full rescue of the *lrx1* single mutant phenotype was observed with all three constructs (S7B Fig). By contrast, only *L10E1* complemented the *lrx1 lrx2* double mutant phenotype to the same extent as *L1E1*, *L3E1*, *L4E1*, or *L5E1* (Fig 6A). To quantify the extent of LRX protein activity, root hair density and root hair length were determined. Only trichoblast cells with root hair structures not limited to initial bulge formation were considered for the measurements. The *lrx1 lrx2* double mutant forms very few root hair structures, which is reflected by a very low root hair density compared to the wild type. Seedlings expressing *L8E1* or *L11E1* displayed only partial, yet significant alleviation of this *lrx1 lrx2* mutant phenotype (Fig 6B). The length of the root hair structure is strongly reduced in the *lrx1 lrx2* mutant, and this phenotype is also less alleviated in *L8E1* and *L11E1* expressing lines compared to the other lines (Fig 6C). Together, these results suggest that the NT- and LRR-domains of different LRX proteins of vegetative tissues are sufficiently overlapping in their activities to replace *LRX* genes active in other vegetative cell types. By contrast, pollen-expressed *LRX*s have functionally diverged to varying degrees, some being similar but not equivalent to the *LRX*s expressed in vegetative tissues.

### LRX downstream signaling components

**Fig 7:**
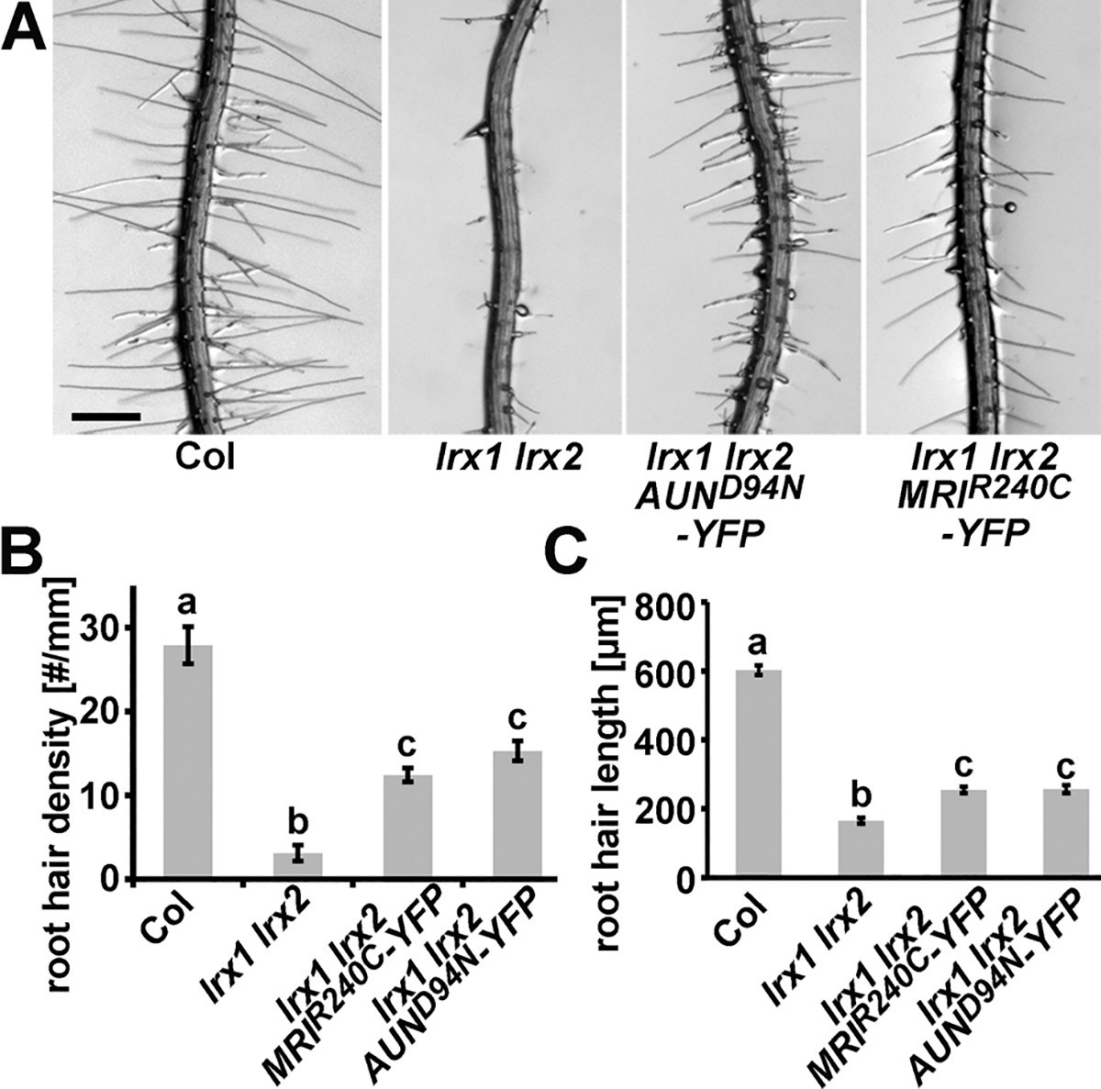
Expression of *AUN1^D94N^* and *MRI^R240C^* partially suppress the *lrx1 lrx2* root-hair defect. (A) Root hair phenotypes of the wild type (Col), *lrx1 lrx2* double mutant, and representative examples of several independent transgenic *lrx1 lrx2* double mutant expressing either *pMRI*:*AUN1^D94N^-YFP* or *pMRI*:*MRI^R240C^-YFP*. Seedlings were grown for 5 days on half-strength MS in a vertical orientation. Quantification of the rescue by assessing root hair density (B) and root hair length (C) in the different lines. Seedlings were grown for 5 days on half-strength MS in a vertical orientation. Error bars represent SEM; different letters above the graphs indicate significant differences (T-test, N = 50, P<0.01). Bar = 0.5 mm.

The FER homologs ANX1 and ANX2 are required to maintain pollen tube integrity during growth [6,49] and the type one protein phosphatase *AUN1^D94N^* and the receptor-like cytoplasmic kinase *MRI^R240C^* dominant variants were found to suppress *anx1 anx2* male sterility [28,29]. To test whether the signaling pathways involving CrRLK1L and LRX proteins are comparable in reproductive and vegetative tissues, the *lrx1 lrx2* double mutant was transformed with an *AUN1^D94N^-YFP* and a *MRI^R240C^-YFP* construct under the control of the *MRI* promoter that is particularly active in pollen, and root hairs [28]. Several independent T_2_ plants transgenic for either of the two constructs displayed partial suppression of the *lrx1 lrx2* root hair phenotype (Fig 7A, S8 Fig), which was confirmed by the quantification of root hair density as well as root hair length. The reduced root hair density is partly suppressed by both *AUN1^D94N^-YFP* and *MRI^R240C^-YFP* (Fig 7B), even though wild-type levels are not reached. Measuring root hair length revealed again much shorter root hairs in the *lrx1 lrx2* mutant compared to the wild type, and an intermediate root hair length in the *lrx1 lrx2* lines expressing *AUN1^D94N^-YFP* or *MRI^R240C^-YFP* (Fig 7C). These two constructs were previously shown to induce shorter root hairs in a wild-type background, explaining the lack of full suppression of the *lrx1 lrx2* short root hair phenotype [28,29]. Hence, both AUN1 and MRI play a role in the root hair signaling pathway downstream of LRX1 and LRX2.

## Discussion

In this work, we demonstrate that the capability to interact with FER is not limited to LRX4 as demonstrated earlier [41] but shared by several of the LRX proteins of very different cell types (Fig 2, S1 Fig), indicative of an active LRX-FER interaction system being generally relevant for cell growth and CWI sensing [30,40,41,42]. This assumption is corroborated by similar phenotypes of *fer-4* and an *lrx12345* quintuple mutant (Fig 3). Comparable to the interaction with FER, several LRXs including LRX1 interact with RALF1 (Fig 2, Table 1, S2 Fig). These interactions appear not to be mutually exclusive as a negative impact of RALF1 peptide on LRX1-FER interaction was not observed (Fig 2). RALF-LRX and RALF-FER interaction studies show that LRXs and FER bind the C-terminal half of RALF peptides [27,50,51]. A simultaneous binding of LRXs and FER to the same RALF peptide, however, appears unlikely. Yet, in the recently described RALF23-LLG2-FER trimeric complex, residues in the RALF23 N-terminal half interact with FER while the C-terminal half of RALF23 could not be located in the crystallization study [27]. It cannot be excluded that upon binding to the LRR-domain of LRXs, RALF peptides induce the interaction of LRXs with FER, without being themselves involved in direct interaction with both LRXs and FER. This would imply that the binding site of RALFs and FER on LRXs are different. While we have observed interaction of FER^ECD^ with the LRR-domain of several LRXs in yeast-two-hybrid experiments, i.e. in the absence of RALFs (S1 Fig), RALF peptides might have a significant influence on this interaction *in planta*. It is possible that RALFs present in tobacco support the formation of the LRX1-FER interaction observed in Co-IP experiments and additional RALF1 does not further improve the formation of this protein complex. While the amino acid residues of the LRR domain involved in LRX-RALF interaction have been identified for some LRX/RALF pairs [51], the details on the LRX-FER interaction requires further analyses.

Different LRXs bind an overlapping but not identical array of RALF peptides [40,41], and the full binding spectrum of the different LRXs might be even broader. One reason for the distinct RALF binding spectrum of LRXs is based on their expression pattern. Pollen-localized LRX8 shows a much higher affinity to the pollen-localized RALF4 than the root/shoot-localized RALF1 [42]. It can be speculated that the diverse affinities of RALFs and LRXs contribute to the specificities of the plant’s response to different RALF peptides that have distinct biological activities [17,18,19,20,26,52]. An additional regulatory layer is added by the pH that is influenced by e.g. RALFs and modifies their binding to interaction partners as demonstrated for the CrRLK1L THE1 and its interaction partner RALF34 [20]. Similarly, binding of RALF4 to LRX8 is pH-dependent and stronger under acidic conditions [51]. *In vivo* and *in vitro* analyses have shown that dimerization of LRXs is necessary for biological activity [51]. Whether LRXs form homo- and/or heterodimers *in vivo* and whether these can bind different RALFs remains to be unraveled. Possibly, the exact pairs of LRX-RALF interactions influence binding to other proteins, similar to RALF23 that modifies the scaffolding function of FER inducing the dissociation of FLS2 from BAK1 [17]. Clearly, LRXs do not only interact with FER and RALFs. The data presented here reveal that the previously demonstrated membrane association of LRX4^ΔE^ [37] requires the LRR-domain but is independent of FER as LRX4^ΔE^ is also found in the membrane fraction of a *fer-4* mutant (Fig 1). Hence, LRXs might also bind other CrRLK1Ls or other plasma membrane-associated receptors. BAK1 or LLG1/2 are both required for RALF perception, interact with FER, and are in or at the plasma membrane, respectively [26,27]. In a recent work, however, the authors did not observe interaction of LRX8 with LLGs [51], making this interaction less likely.

While there is increasing understanding of the role of the LRR-domain of LRXs, the role of the NT-domain remains largely elusive. Its function is not related to the membrane association of LRXs, as the LRX4^ΔEΔNT^ still associates with the plasma membrane (Fig 1) and is dispensable for interaction with RALFs or FER (S1 Fig, S2 Fig). The NT-domain is only found in LRX-type proteins [30] and is important for their activity, since deletion of the NT-domain in LRX1 (LRX1^ΔNT^) impairs protein function. The inconsistent phenotypes observed in the *lrx1* mutant complemented with LRX1^ΔNT^ (Fig 4) suggest that the NT-domain possibly has a stabilizing function or undergoes interaction(s) with binding partners that increase protein activity. The Cys-rich CRD domain linking the LRR- and the extensin-domain [30] folds back onto the LRR domain and forms several di-sulfide bridges [51]. While these might stabilize the LRR domain, they are not essential for binding to FER, since the CRD is not present in the yeast-two-hybrid proteins (S1 Fig), is not directly involved in RALF binding [51], and not required for the dominant-negative effect of LRX1^ΔE^ [33].

Earlier experiments with the root hair-expressed *LRX1* and *LRX2* suggested synergistic interaction and functional equivalence of these two LRXs, with the *lrx1 lrx2* double mutant developing a *fer-4*-like root hair defect ([35] and Fig 3). The rescue experiments for *lrx1*, *lrx1 lrx2*, and *lrx345* mutants suggest that the NT- and LRR-domains of LRXs of vegetative tissues are functionally similar (Fig 6, S7 Fig). Effective complementation by L3E1 is interesting as it appears to bind RALF1 with lower affinity than other LRXs (Table 1). The binding of other RALFs by LRX3 [40], in combination with the overexpression by the constitutively active *35S* promoter, might be sufficient for its FER-related activity.

The overexpression of chimeric *LRX8* and *LRX11* (as *L8E1* and *L11E1* fusion constructs) failed to fully complement the *lrx1 lrx2* mutant (Fig 6). This divergence is supported by phylogenetic analyses that revealed evolutionary separation of pollen- and vegetatively expressed LRXs [47,48], suggesting that the NT- and LRR-domains of LRX8 and LRX11 have too strongly diversified to fulfill the same functions as the ‘vegetative’ LRXs analyzed here. The homology of these domains in LRX8, LRX10, and LRX11 compared to LRX1 are similar with around 50% identity [47], revealing that LRX10 is not particularly more similar to LRX1 than LRX8 and LRX11. In pollen tubes, expression of *AUN1^D94N^*, a dominant amorphic variant of AUN1, partially suppresses not only the *anx1 anx2* but also the *lrx8-11* quadruple mutant pollen bursting phenotype [29]. This is indicative of LRX8-11 and ANX1/ANX2 being active in the same pathway. The *FER*-homologs *ANX1*/*2* and *BUPS1/2* but not *FER* are expressed in pollen tubes [6,10,21] and pollen-expressed LRXs possibly function in conjunction with these CrRLK1Ls. The finding was therefore not unexpected that *AUN1^D94N^* alleviates the *lrx1 lrx2* mutant root hair phenotype. Interestingly, the *anx1 anx2* suppressor mutant *MRI^R240C^* also affects the *lrx1 lrx2* root hair phenotype (Fig 7), even though it does not suppress the pollen tube defect of the *lrx8-11* mutant [29]. Hence, the LRX-RALF-CrRLK1L signaling modules of diverse cell types involve at least partially conserved downstream components.

Potential differences in the function of the LRX extensin domains have not been investigated here, but rather avoided by using the *LRX1* extensin coding sequence for all chimeric complementation constructs. The extensin domain is variable among the LRXs both in terms of length and the repetitive motifs typical for this structural protein domain [32,47]. Likely, the extensin domains of the different LRXs have adapted to the specific cell wall composition of the various tissues they are active in, which would explain the considerable differences in the extensin domains [47]. Extensins form covalent links with other extensins or with polysaccharides in the cell wall [53,54,55], and the composition of cell walls differs considerably among cell types [56,57].

Cell growth requires the controlled simultaneous expansion of the cell wall and the protoplast. The LRX-FER binding described here is a possible way to coordinate the enlargement of the two cellular compartments, since the interaction partners are attached/embedded in their respective subcellular structure. A potential function of LRXs may be to stabilize FER in its activity to regulate cell growth and possibly also as a scaffolding protein controlling immune signaling [17]. In addition to FER and RALF1 used in this study, different RALFs and probably different CrRLK1Ls are possible binding partners of LRXs and the specificities of interactions might reflect differences in the biological processes they are involved in.

## Materials and Methods

### Plant growth and propagation

*Arabidopsis thaliana* of the ecotype Columbia were used for all experiments. Seeds were surface sterilized with 1% Sodium hypochlorite, 0.03% TritonX-100, washed three times with sterile water, and, unless stated otherwise, plated on half-strength MS plates (0.5X MS salt, 2% sucrose, 0.5 mg/L nicotinic acid, 0.5 mg/L pyridoxine-HCl, 0.1 mg/L thiamine-HCl, glycine 2 mg/L, 0.5 g/L MES, pH 5.7, 0.6% Gelzan (Sigma); referred to as standard medium) under a 16 hrs light– 8 hrs dark photoperiod at 22°C. For propagation and crossings, plants were grown under the same conditions in soil.

For selection of transgenic lines, seeds of plants used for Agrobacterium (GV3101)-mediated transformation by the floral-dip method, were plated on standard medium containing 0.8% bactoagar, supplemented with appropriate antibiotics; 100 μg/ml ticarcillin, 50 μg/ml kanamycin or 10 μg/ml glufosinate-ammonium.

### Molecular markers

PCR-based molecular markers used to produce the different lines are described in [36] for *lrx3*, *lrx4*, and *lrx5*, [58] for *lrx1*, [35] for *lrx2*, and [41] for *fer-4*. The primers used for the PCR reactions are listed in the S2 Table.

### DNA constructs

For the *LRX4^ΔE^* construct, the coding sequence of the N-terminal half of LRX4 was amplified using the primers LRX4oE_XhoI_F and LRX4_PstI_R (S3 Table). This product was digested with *Xho*I and *Pst*I and ligated with a fragment encoding a double FLAG tag with a *Pst*I and a *Xba*I site at the 5’ and 3’ end, respectively, into the plasmid pART7 [59] digested with *Xho*I and *Xba*I, resulting in the *35S*:*LRX4^ΔE^-2FLAG* construct. All final constructs were sequenced. The *35S*:*LRX4^ΔE^-Citrine* construct containing the identical *LRX4^ΔE^* sequence fused to *Citrine* is described elsewhere [41].

For *LRX3^ΔE^-FLAG*, the construct *LRX4^ΔE^-FLAG* was cut with *Kpn*I, *Pst*I, and the *LRX4^ΔE^* sequence replaced by *LRX3^ΔE^*, which was amplified using the oligos LRX3_ KpnI_F and LRX3_PstI_R and cut with *Kpn*I, *Pst*I.

For *LRX1^ΔE^ -FLAG*, the *LRX1* fragment was amplified using the primers LRX1_XhoI_F and LRX1_PstI_R, digested with *Xho*I/*Pst*I and cloned into the vector *pART7_LRX4^ΔE^-FLAG* digested with the same enzymes to release the *LRX4^ΔE^* sequence. For the *35S*:*L1E1* construct, the plasmid *35S*:*LRX1^ΔE^-2FLAG* was opened with *Pst*I and *Xba*I and a *Pst*I-*Spe*I fragment containing the extensin-coding sequence [35] was inserted.

The *LRX1*:*LRX1^ΔE^* construct containing the *cMyc* tag in front of the *LRR* domain is described elsewhere [34]. For the *LRX1*:*LRX1^ΔLRRΔE^* construct, the promoter and coding sequence up to the end of the cMyc-tag was amplified with the primers LRX1_Prom1000_F and LRX1_ΔLRR_SpeI_R, and the resulting fragment was digested with *Mlu*I (in the *LRX1* promoter) and *Spe*I (at the end of the myc tag sequence) and cloned into the *LRX1*:*LRX1* construct cut with the same enzymes (*Spe*I overlapping with the stop codon of the *LRX1* coding sequence). For *LRX1*:*LRX1^ΔNTΔE^*, the promoter and signal peptide coding sequence was amplified with the primers LRX1_Prom1000_F and LRX1_ΔNT_SaII_R and the resulting fragment was digested with *Mlu*I (in the promoter) and *Sal*I (at the end of the signal peptide sequence) and cloned into *LRX1*:*LRX1^ΔE^* cut with the same enzymes (the *Sal*I site in the *LRX1*:*LRX1^ΔE^* construct is at the beginning of the *cMyc* coding sequence). For *LRX1*:*LRX1^ΔNT^* the *Mlu*I-*Sal*I fragment of *LRX1*:*LRX1^ΔNTΔE^* was ligated into *LRX1*:*LRX1* cut with the same enzymes.

For the *35S*:*L3/4/5/8/10/11-E1* constructs, the coding sequences from the ATG to the CRD-coding sequence were amplified with primers (S3 Table) introducing a *Kpn*I or an *Xho*I and a *Pst*I site at the 5’ and 3’ end of the PCR product, respectively, and the fragments were ligated into *35S*:*L1E1* cut with the same enzymes to release the L1 coding sequence.

All the *pART7*-based expression cassettes were cut out with *Not*I and cloned into the binary vector *pART27* [59] cut with the same enzyme.

Cloning of *MRI^R240C^* CDS without stop codon in Gateway compatible binary vector *pMRI*:*-*:*GW-YFP* plasmid (*pABD83*, Basta Resistance) to obtain *pMRI*:*MRI^R240C^-YFP* was described previously [28]. To obtain *pMRI*:*AUN1^D94N^-YFP*, *AUN1^D94N^* CDS without stop codon in *pDONR207* (Invitrogen) [29] was remobilized into *pABD83*.

The *BD-LRX4* and *AD-FER^ECD^* constructs for the Yeast-two-hybrid experiment were cloned as previously described [41], where *NtermFER* equals *AD-FER^ECD^* and *LRR4* equals *BD-LRX4*. For the *BD-LRX1/2/3/5* constructs, the coding sequence of the LRR domain of the *LRXs* was amplified using primers (S3 Table) to introduce a *Bam*HI and a *Xho*I site at the 5’ and 3’ end of the PCR fragments, respectively. These were cloned into *pJET1*.*2* (Thermo Scientific) and correct clones were cut with *Bam*HI and *Xho*I and ligated into *pGBKT7* cut with *Bam*HI and *Sal*I. The AD-RALF1 construct was cloned into *pJET1*.*2* (Thermo Scientific) by amplification of the coding sequence with the primers y2h_RALF1_F and y2h_RALF1_R. A correct clone was cut with *Eco*RI and *Xma*I and ligated into *pGADT7* cut with *Eco*RI and *Xma*I.

### Phenotyping of seedling growth properties

For the quantification of gravitropism, seedlings were grown in a vertical orientation on standard MS medium for 8 days, and the ratio of root progression in the vertical axes over total root length was used as the parameter, as described [46]. For measurements, the plates were scanned and analyzed by ImageJ. To ascertain consistent results, seedlings of different generations were used and at least 10 seedlings were measured for one data point. The numerical data underlying these and all other graphs presented are provided as supplementary data.

The accumulation of anthocyanin was quantified on 12 days-old seedlings grown in a vertical orientation on standard medium by published methods [60,61]. Twenty seedlings were pooled and incubated in 45% Methanol, 5% acetic acid. After centrifugation for 5 min at RT and 13’000 rpm, the supernatant was used to measure absorption at 530 nm for anthocyanin and at 657 nm for chlorophyll content correction; final value = Abs530nm-(0.25xAbs657nm). One data point in the graph is the average of quadruplicates.

For root length measurements, seedlings were grown for 5 days on standard medium in a vertical orientation, plates were scanned, and ImageJ was used to measure root length. The average of at least 15 seedlings was used for one data point.

Root hair phenotypes were assessed in 5 days-old seedlings grown in a vertical orientation on standard medium. Pictures of root hair were taken with a MZ125 Binocular (Leica), using a DFC420 digital camera (Leica).

### Co-immunoprecipitation

For pulldown and Co-IP analysis of the different constructs indicated in the experiments DNA was infiltrated into *Nicotiana benthamiana* leaves, and after 48 hrs, the leaves were excised and ground in liquid nitrogen as described in [62]. Two hundred mg of tissue powder was re-suspended in 500 μL extraction buffer [200 mM Tris-HCl (pH 7.5), 150 mM NaCl, 1 mM PMSF, protease inhibitor and 0.5% Triton X-100]. The suspension was incubated on ice for 30 minutes and then centrifuged at 11,000 rpm for 20 minutes at 4°C. The supernatant (200 μL) was then incubated with 10 μL anti-HA (Thermo Scientific, 88837), or anti-FLAG magnetic beads overnight at 4°C on a rotating shaker. If RALF1 peptide was added, the peptide was diluted in such way that for every sample 1μL of peptide was added to the protein extracts. After incubation, the beads were washed five times with the wash buffer (extraction buffer containing 0.05% Triton X-100) and boiled in SDS-PAGE loading buffer for 15 minutes at 75°C. The immunoprecipitates were then run on an SDS-PAGE gel and transferred to nitrocellulose membrane to perform immunoblotting using the following antibodies: anti-FLAG M2 (Sigma-Aldrich, F3165), anti-GFP (Biolegend, 902601), anti-HA-HRP 3F10 (Roche, 12 013 819 001) and anti-mouse-HRP (Sigma Aldrich, A 4416). For Co-IP experiments with LRX1/FER and LRX3/FER, the sensitivity of the immunoblotting was augmented with the Western Blot Enhancer kit (ThermoFisher).

### BLITZ analysis

The BLITZ experiments were performed as previously described [42]. The *LRR^ΔE^*–FLAG versions of the different *LRXs* were expressed using the *35S* promoter in *N*. *benthamiana*, presence of proteins was checked by immunoblotting, and proteins were immune-precipitated as described above. After immunoprecipitation, elution was performed with 30 μL of 1M Glycine (pH 2.0) buffer for 2 min in a Thermomixer (Eppendorf) at 1200 rpm, then beads were spun down for 2 min at 1300 x g at RT, and the supernatant was neutralized with 30 μL of 1M Tris-HCl (pH 9.5). Protein concentration was determined by Qubit measurement (Quant-iTTM Protein Assay kit, Invitrogen). Samples were diluted 1:1 with sample diluent buffer (Pall FortéBio cat18-1091) to a concentration of 0.142 mg/ml for analysis using the BLITZ system. The same buffer was used to dilute the anti-FLAG M2 antibody (Sigma-Aldrich) 1:50 to a final concentration of 4 μg/ml. A 1:1 mix of sample:antibody was then incubated for 30 minutes at RT, and loaded onto the protein A biosensor (Pall FortéBio cat 18–5010). The experiment was divided into 5 different steps: Initial baseline duration (30 s), Loading duration (120 s), Baseline duration (30 s), Association duration (120 s), and Dissociation duration (120 s). Different RALF synthetic peptide (PHTD peptides) concentrations (200 μM, 150 μM, 100 μM, 50 μM, 20 μM, 15 μM, 10 μM, 4 μM, 2 μM and 0.2 μM) were added to quantify the protein interaction.

### Immunoblotting

To test the accumulation of LRX1^ΔE^, LRX1^ΔLRRΔE^, and LRX1^ΔNTΔE^ proteins, root material of 300 seedlings grown for 10 days in a vertical orientation was collected and ground in liquid nitrogen. Around 50 mg of fresh material was extracted with 200 μL 0.1% SDS by vortexing, immediately followed by heating to 95°C for 5 min. After cooling, material was centrifuged at 13’000 rpm for 10 min and 20 μL of the supernatant was used for SDS-PAGE and blotting to nitrocellulose membranes using semi-dry blotting. After over-night blocking of the membranes in 1xTBS, 0.1% Tween-20, 5% low-fat milk powder, the membranes were incubated in 1xTBST (1xTBS, 0.1% Tween-20), 0.5% low-fat milk powder containing primary antibodies as indicated in the figures, followed by a peroxidase-coupled secondary antibody, diluted 1:3000 each. After each antibody incubation, the membranes were washed three times for 10 minutes with 1xTBST. The signal of the secondary antibody was detected using the WesternBright ECL kit (Advansta, K-12045-D20) with the Fusion FX fusion imager (Vilber).

### RT-PCR

Semi-quantitative RT-PCR was performed on RNA isolated of 10 days-old seedlings using the total RNA isolation system (Promega). Reverse transcription was performed on 300 ng of total RNA using the iScript advanced kit (BioRad). PCR was performed using gene-specific primers as listed in the S4 Table. Correct amplification of the expected DNA band was verified by sequencing of the PCR products.

### Yeast-two-hybrid

Transformation of the yeast strain PJ69-4A [63] was done following standard procedures and quadruple drop-out medium lacking Leu, Trp, His, and Ade were used to screen for positive interactions after 4 days incubation at 30°C. Three different colonies containing both vectors were mixed and plated in triplicates on quadruple drop-out medium.

### Membrane fractionation

Membrane fractionation was performed as described [37] using an established method [64]. Homogenized tissue samples were suspended in 3 volumes of ice-cold extraction buffer [250 mM sorbitol; 50 mM Tris-HCl, 2 mM EDTA; pH 8.0 (HCl); immediately before use add: 5 mM DTT; 0.6% insoluble PVP; 1 mM PMSF; 10 μL/mL Protease Inhibitor Cocktail (Sigma P9599)]. The material was first centrifuged in an Eppendorf centrifuge (ThermoScientific) at 5,000g and 8,000g for 5 minutes each at 4°C to remove cell debris. The supernatant was then centrifuged at 40,000 rpm for 1 hour at 4°C in an Optima XPN-100 ultracentrifuge (Beckman Coulter) using a SW41-Ti swing-out rotor, the supernatant removed to 300 uL, and 30 uL of 10% SDS was added to the pelleted membrane fraction for protein solubilization. The samples were used for SDS-PAGE and immunoblotting, where the LRX4^ΔE^-FLAG, LRX4^ΔE^-Citrine, and the marker proteins were detected with the antibodies indicated.

### Gene identifiers of genes used in this study

FER: At3G51550; RALF1:At1G02900; LRX1: At1g12040; LRX2: At1g62440; LRX3: At4g13340; LRX4: At3g24480; LRX5: At4g18670; LRX8: At3g19020; LRX10: At2g15880; LRX11: At4g33970

## Supporting information

S1 Fig Interaction of LRXs and FER in yeast-two-hybrid assays. The yeast strain PJ69-4A was transformed with *pGADT7-FER*^*ECD*^ and *pGBKT7-LRRs* (encoding the LRR domain of the different LRXs as indicated), and with either of the constructs with empty plasmids for the negative controls. The clear difference in yeast growth under selective conditions (-Trp, Leu, His, Ade drop-out medium containing 5 mM 3-AT) indicates interaction of the LRX proteins with FER.
(PDF)

S2 Fig Interaction of LRX1 and RALF1 in yeast. The yeast strain PJ69-4A was transformed with *pGADT7-RALF1* and *pGBKT7-LRR1* or *pGBKT7-LRR4*, and with either of the constructs with empty plasmids for the negative controls. The clear difference in yeast growth under selective conditions (Trp, Leu, His, Ade drop-out medium) indicates interaction of the LRX proteins with RALF1.
(PDF)

S3 Fig Synthetic RALF1 peptide activity. Arabidopsis seedlings were grown for five days in liquid medium in the presence of 1 μM RALF1, which significantly inhibited root growth compared to the control plants grown without RALF1 (student T-test; n = 6; p<0.001). Error bars represent standard error of the mean.
(PDF)

S4 Fig BLITZ output data. Tobacco expressed and immunoprecipitated LRX4^ΔE^–FLAG is attached to the sensor resulting in a signal for bound protein. After washing to remove excess LRX4^ΔE^–FLAG protein. RALF1 peptide is added in different concentrations, followed by dissociation of RALF1. Association/dissociation or RALF1 depends on the concentration of applied RALF1, based on which the Kd is calculated.
(PDF)

S5 Fig LRX1^ΔNT^ protein levels in transgenic *lrx1* and *lrx1 lrx2* mutants. Root material of 10 days-old seedlings was used to detect *LRX1*^ΔNT^ with the anti-cmyc antibody 9E10. Protein levels in transgenic *lrx1* vary but do not correlate with the inconsistent phenotypes observed in these lines as shown in Fig 4C. Transgenic *lrx1 lrx2* double mutants produce the protein but fail to complement the phenotype as shown in Fig 4C. Arrow indicates expected band of LRX1^ΔNT^, which runs at a much higher molecular weight than the calculated 84 kDa, due to glycosylation of the extensin domain.
(PDF)

S6 Fig RT-PCR confirming expression of the transgenes. Total RNA was extracted from 7-days old seedlings and transgene-specific RT-PCR was performed to confirm expression of the transgenes. In all experiments, *Actin2* was used as endogenous control for comparable amounts of RNA in all samples and absence of contaminating genomic DNA. Genomic DNA of *Actin2* results in a longer PCR product as shown. (A) Expression of *LRX1* in the *lrx345* triple mutant background. (B) and (C): the label *LxE1* referring to the different transgenes as indicated. The lane «Col» represents genomic DNA.
(PDF)

S7 Fig Complementation of *lrx345* and *lrx1* mutants. (A) *lrx345* triple mutant plants are smaller at flowering stage than the wild type (Col). This phenotype is complemented by the *35S*:*LRX1* construct. (B) The *lrx1* root hair defect is complemented by the *LRX* chimeric constructs. Bar: 1cm (A); 0.5 mm (B).
(PDF)

S8 Fig Fluorescence of *AUN1^D94N^-YFP* and *MRI^R240C^-YFP* transgenic lines. Transgenic lines expressing *MRI^R240C^-YFP* or *AUN1^D94N^-YFP* show YFP fluorescence at the plasma membrane/cytoplasm and in the nucleus/cytoplasm, respectively. Individual root hairs (A) and entire roots (B) are shown. (B) When expressing *MRI^R240C^-YFP*, root hair growth is frequently arrested resulting in shorter root hairs (yellow arrows) whereas others grow normally (green arrows). Some root hairs still burst after some time (white arrow). Bar = 20 μm (A), 50 μm (B).
(PDF)

S1 Table Molecular weight of recombinant proteins used in this study. (PDF)

S2 Table Primers used for genotyping. (PDF)

S3 Table Primers used for cloning. (PDF)

S4 Table Primers used for RT-PCR. (PDF)

S1 Data Numerical data underlying all graphs shown in figures. (XLSX)
