# Supplementary material for: Overlapping functions and protein-protein interactions of LRR-extensins in Arabidopsis
Source: PLoS Genet. 2020 Jun 19;16(6):e1008847. doi: 10.1371/journal.pgen.1008847 (PMC7357788; doi:10.1371/journal.pgen.1008847)
Supplement: S1 Fig — The yeast strain PJ69-4A was transformed with pGADT7-FERECD and pGBKT7-LRRs (encoding the LRR domain of the different LRXs as indicated), and with either of the constructs with empty plasmids for the negative controls. The clear difference in yeast growth under selective conditions (-Trp, Leu, His, Ade drop-out medium containing 5 mM 3-AT) indicates interaction of the LRX proteins with FER. (PDF) [file pgen.1008847.s001.pdf]

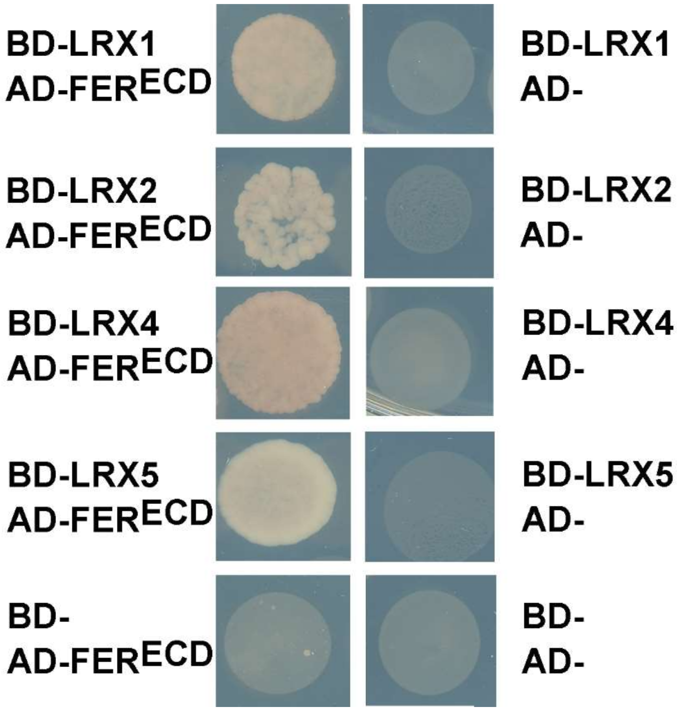

S1 Fig Interaction of LRXs and FER in yeast-two-hybrid assays.
The yeast strain PJ69-4A was transformed with *pGADT7-FER^{ECD}* and *pGBKT7-LRRs* (encoding the LRR domain of the different LRXs as indicated), and with either of the constructs with empty plasmids for the negative controls. The clear difference in yeast growth under selective conditions (-Trp, Leu, His, Ade drop-out medium containing 5 mM 3-AT) indicates interaction of the LRX proteins with FER.
