# Supplementary material for: Overlapping functions and protein-protein interactions of LRR-extensins in Arabidopsis
Source: PLoS Genet. 2020 Jun 19;16(6):e1008847. doi: 10.1371/journal.pgen.1008847 (PMC7357788; doi:10.1371/journal.pgen.1008847)
Supplement: S2 Fig — The yeast strain PJ69-4A was transformed with pGADT7-RALF1 and pGBKT7-LRR1 or pGBKT7-LRR4, and with either of the constructs with empty plasmids for the negative controls. The clear difference in yeast growth under selective conditions (Trp, Leu, His, Ade drop-out medium) indicates interaction of the LRX proteins with RALF1. (PDF) [file pgen.1008847.s002.pdf]

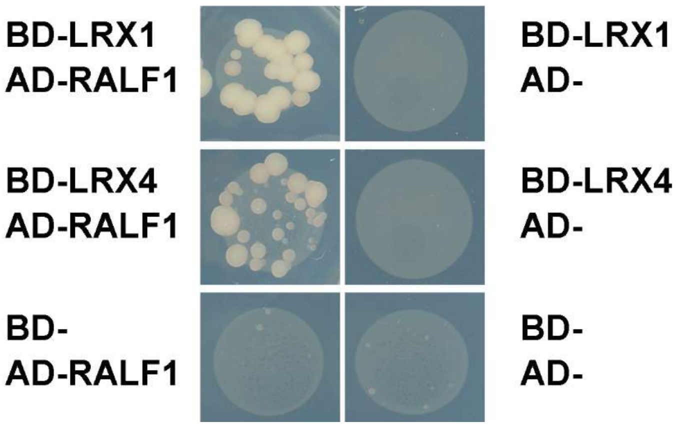

S2 Fig Interaction of LRX1 and RALF1 in yeast.

The yeast strain PJ69-4A was transformed with *pGADT7-RALF1* and *pGBKT7-LRR1* or *pGBKT7-LRR4*, and with either of the constructs with empty plasmids for the negative controls. The clear difference in yeast growth under selective conditions (Trp, Leu, His, Ade drop-out medium) indicates interaction of the LRX proteins with RALF1.
