# Supplementary material for: Overlapping functions and protein-protein interactions of LRR-extensins in Arabidopsis
Source: PLoS Genet. 2020 Jun 19;16(6):e1008847. doi: 10.1371/journal.pgen.1008847 (PMC7357788; doi:10.1371/journal.pgen.1008847)
Supplement: S3 Fig — Arabidopsis seedlings were grown for five days in liquid medium in the presence of 1 μM RALF1, which significantly inhibited root growth compared to the control plants grown without RALF1 (student T-test; n = 6; p<0.001). Error bars represent standard error of the mean. (PDF) [file pgen.1008847.s003.pdf]

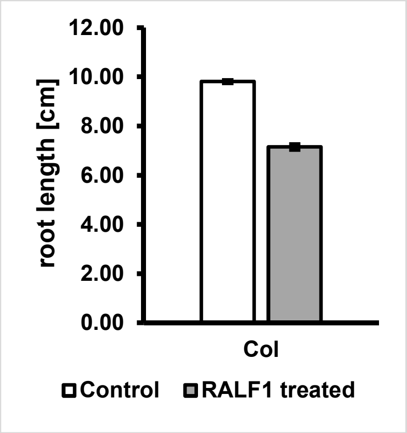

S3 Fig Synthetic RALF1 peptide activity.

Arabidopsis seedlings were grown for five days in liquid medium in the presence of 1 μM RALF1, which significantly inhibited root growth compared to the control plants grown without RALF1 (student T-test; n=6; $p<0.001$). Error bars represent standard error of the mean.
