# Supplementary material for: Overlapping functions and protein-protein interactions of LRR-extensins in Arabidopsis
Source: PLoS Genet. 2020 Jun 19;16(6):e1008847. doi: 10.1371/journal.pgen.1008847 (PMC7357788; doi:10.1371/journal.pgen.1008847)
Supplement: S4 Fig — Tobacco expressed and immunoprecipitated LRX4ΔE–FLAG is attached to the sensor resulting in a signal for bound protein. After washing to remove excess LRX4ΔE–FLAG protein. RALF1 peptide is added in different concentrations, followed by dissociation of RALF1. Association/dissociation or RALF1 depends on the concentration of applied RALF1, based on which the Kd is calculated. (PDF) [file pgen.1008847.s004.pdf]

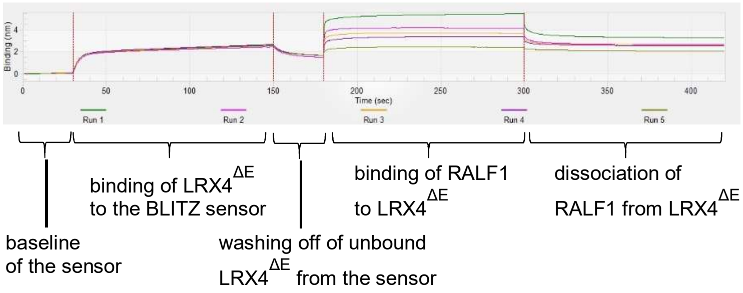

S4 Fig BLITZ output data.

Tobacco expressed and immunoprecipitated LRX4$^{\Delta E}$–FLAG is attached to the sensor resulting in a signal for bound protein. After washing to remove excess LRX4$^{\Delta E}$ –FLAG protein. RALF1 peptide is added in different concentrations, followed by dissociation of RALF1. Association/dissociation or RALF1 depends on the concentration of applied RALF1, based on which the Kd is calculated.
