# Supplementary material for: Overlapping functions and protein-protein interactions of LRR-extensins in Arabidopsis
Source: PLoS Genet. 2020 Jun 19;16(6):e1008847. doi: 10.1371/journal.pgen.1008847 (PMC7357788; doi:10.1371/journal.pgen.1008847)
Supplement: S5 Fig — Root material of 10 days-old seedlings was used to detect LRX1ΔNT with the anti-cmyc antibody 9E10. Protein levels in transgenic lrx1 vary but do not correlate with the inconsistent phenotypes observed in these lines as shown in Fig 4C. Transgenic lrx1 lrx2 double mutants produce the protein but fail to complement the phenotype as shown in Fig 4C. Arrow indicates expected band of LRX1ΔNT, which runs at a much higher molecular weight than the calculated 84 kDa, due to glycosylation of the extensin domain. (PDF) [file pgen.1008847.s005.pdf]

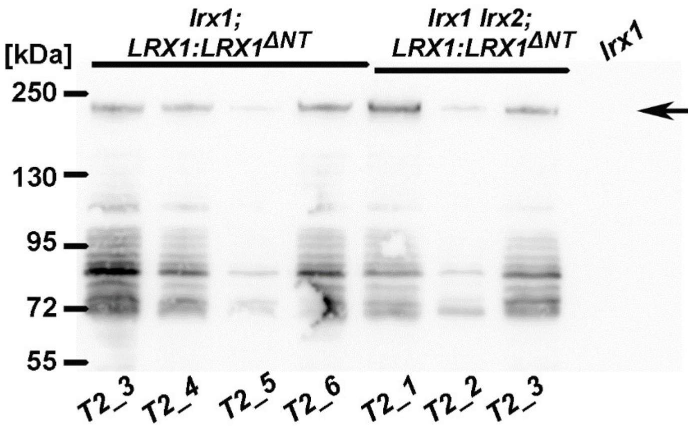

S5 Fig LRX1$^{\Delta NT}$ protein levels in transgenic *lrx1* and *lrx1 lrx2* mutants.

Root material of 10 days-old seedlings was used to detect *LRX1*$^{\Delta NT}$ with the anti-cmyc antibody 9E10. Protein levels in transgenic *lrx1* vary but do not correlate with the inconsistent phenotypes observed in these lines as shown in Fig 4C. Transgenic *lrx1 lrx2* double mutants produce the protein but fail to complement the phenotype as shown in Fig 4C. Arrow indicates expected band of LRX1$^{\Delta NT}$, which runs at a much higher molecular weight than the calculated 84 kDa, due to glycosylation of the extensin domain.
