# Supplementary material for: Overlapping functions and protein-protein interactions of LRR-extensins in Arabidopsis
Source: PLoS Genet. 2020 Jun 19;16(6):e1008847. doi: 10.1371/journal.pgen.1008847 (PMC7357788; doi:10.1371/journal.pgen.1008847)
Supplement: S6 Fig — Total RNA was extracted from 7-days old seedlings and transgene-specific RT-PCR was performed to confirm expression of the transgenes. In all experiments, Actin2 was used as endogenous control for comparable amounts of RNA in all samples and absence of contaminating genomic DNA. Genomic DNA of Actin2 results in a longer PCR product as shown. (A) Expression of LRX1 in the lrx345 triple mutant background. (B) and (C): the label LxE1 referring to the different transgenes as indicated. The lane «Col» represents genomic DNA. (PDF) [file pgen.1008847.s006.pdf]

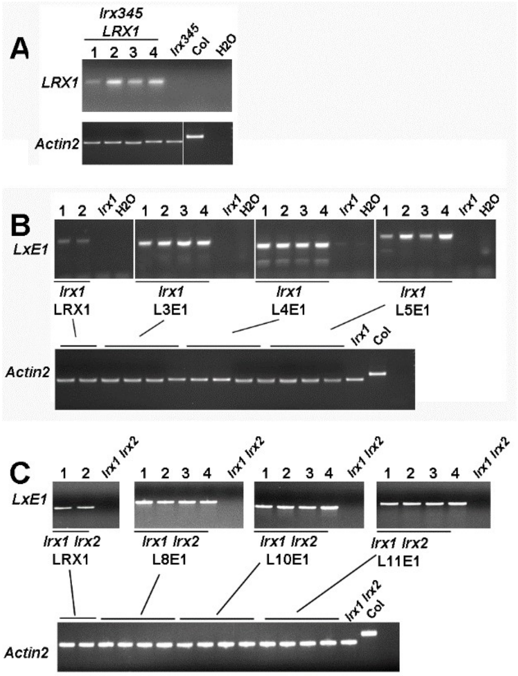

S6 Fig RT-PCR confirming expression of the transgenes.

Total RNA was extracted from 7-days old seedlings and transgene-specific RT-PCR was performed to confirm expression of the transgenes. In all experiments, *Actin2* was used as endogenous control for comparable amounts of RNA in all samples and absence of contaminating genomic DNA. Genomic DNA of *Actin2* results in a longer PCR product as shown. (A) Expression of *LRX1* in the *lrx345* triple mutant background. (B) and (C): the label *LxE1* referring to the different transgenes as indicated. The lane «Col» represents genomic DNA.
