# Supplementary material for: Overlapping functions and protein-protein interactions of LRR-extensins in Arabidopsis
Source: PLoS Genet. 2020 Jun 19;16(6):e1008847. doi: 10.1371/journal.pgen.1008847 (PMC7357788; doi:10.1371/journal.pgen.1008847)
Supplement: S7 Fig — (A) lrx345 triple mutant plants are smaller at flowering stage than the wild type (Col). This phenotype is complemented by the 35S:LRX1 construct. (B) The lrx1 root hair defect is complemented by the LRX chimeric constructs. Bar: 1cm (A); 0.5 mm (B). (PDF) [file pgen.1008847.s007.pdf]

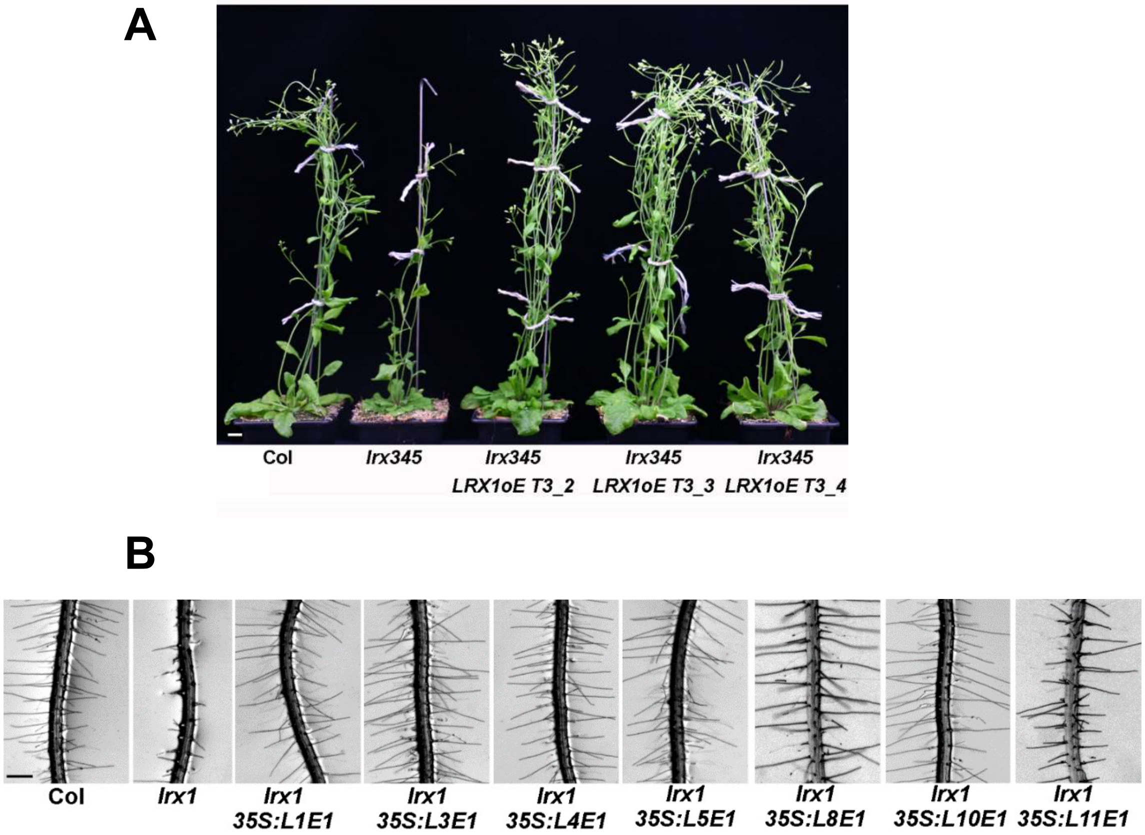

S7 Fig Complementation of *lrx345* and *lrx1* mutants.

(A) *lrx345* triple mutant plants are smaller at flowering stage than the wild type (Col). This phenotype is complemented by the *35S:LRX1* construct. (B) The *lrx1* root hair defect is complemented by the *LRX* chimeric constructs. Bar: 1cm (A); 0.5 mm (B).
