# Supplementary material for: Overlapping functions and protein-protein interactions of LRR-extensins in Arabidopsis
Source: PLoS Genet. 2020 Jun 19;16(6):e1008847. doi: 10.1371/journal.pgen.1008847 (PMC7357788; doi:10.1371/journal.pgen.1008847)
Supplement: S8 Fig — Transgenic lines expressing MRIR240C-YFP or AUN1D94N-YFP show YFP fluorescence at the plasma membrane/cytoplasm and in the nucleus/cytoplasm, respectively. Individual root hairs (A) and entire roots (B) are shown. (B) When expressing MRIR240C-YFP, root hair growth is frequently arrested resulting in shorter root hairs (yellow arrows) whereas others grow normally (green arrows). Some root hairs still burst after some time (white arrow). Bar = 20 μm (A), 50 μm (B). (PDF) [file pgen.1008847.s008.pdf]

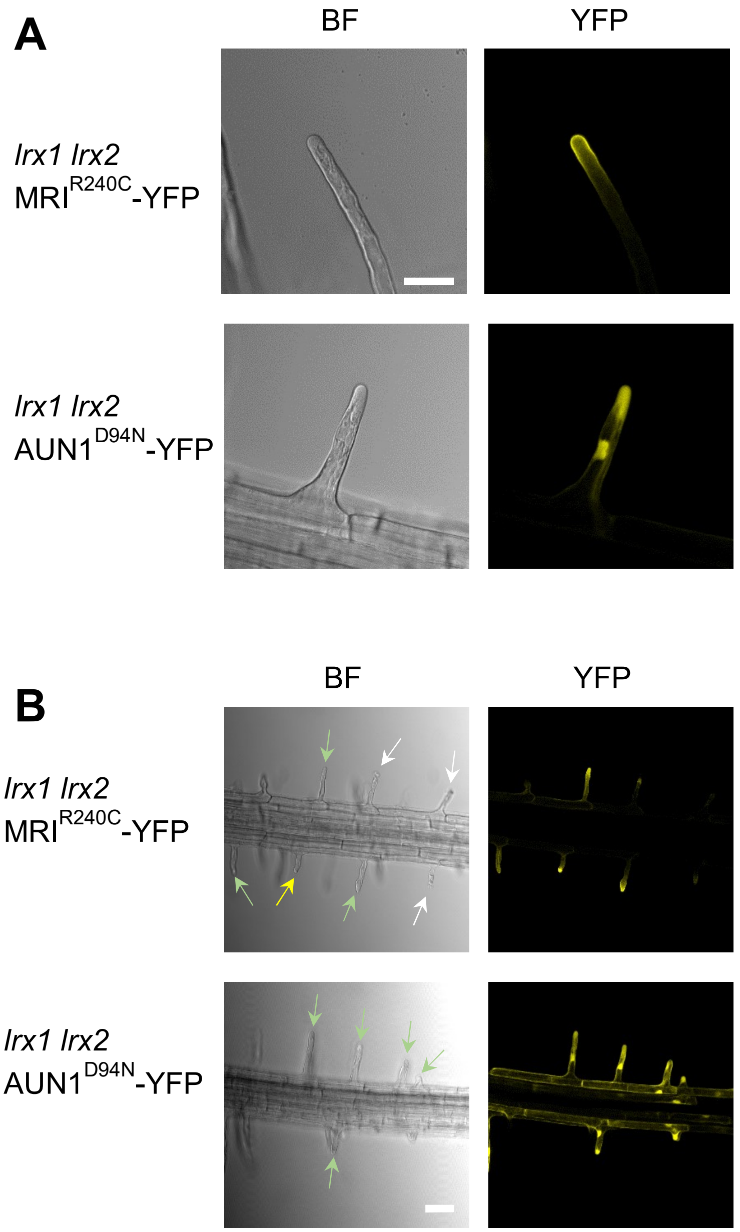

S8 Fig Fluorescence of *AUN1*^{D94N}-*YFP* and *MRI*^{R240C}-*YFP* transgenic lines. Transgenic lines expressing *MRI*^{R240C}-*YFP* or *AUN1*^{D94N}-*YFP* show YFP fluorescence at the plasma membrane/cytoplasm and in the nucleus/cytoplasm, respectively. Individual root hairs (A) and entire roots (B) are shown. (B) When expressing *MRI*^{R240C}-*YFP*, root hair growth is frequently arrested resulting in shorter root hairs (yellow arrows) whereas others grow normally (green arrows). Some root hairs still burst after some time (white arrow). Bar=20 µm (A), 50 µm (B).
