# Supplementary material for: Overlapping functions and protein-protein interactions of LRR-extensins in Arabidopsis
Source: PLoS Genet. 2020 Jun 19;16(6):e1008847. doi: 10.1371/journal.pgen.1008847 (PMC7357788; doi:10.1371/journal.pgen.1008847)
Supplement: S1 Table — (PDF) [file pgen.1008847.s009.pdf]

S1 Table Molecular weight of recombinant proteins used in this study

The molecular weight of the recombinant proteins detected by western blotting are listed. All LRX1 constructs contain a cMyc tag; all other tags are as mentioned in the protein name.

Most of the proteins migrate approximately according to their molecular weight, when compared with the standard PageRuler^{TM}Plus (Thermo Scientific), except RALF1-FLAG and FER-Citrine that both run markedly higher than expected (around 25 kDa instead of 9.5 kDa and around 110 kDa instead of 75.5 kDa, respectively).

recombinant proteins	kDa
LRX1	91.7
LRX1$^{\Delta NT}$	83.9
LRX1$^{\Delta E}$-2FLAG	54.6
LRX1$^{\Delta E}$-Citrine	78.7
LRX1$^{\Delta NT\Delta E}$	46.6
LRX1$^{\Delta LRR\Delta E}$	22.4
LRX3$^{\Delta E}$-2FLAG	45.9
LRX4$^{\Delta E}$-2FLAG	46.9
LRX4$^{\Delta NT\Delta E}$ -2FLAG	37.4
LRX4$^{\Delta LRR\Delta E}$ -2FLAG	16.6
LRX4$^{\Delta E}$-Citrine	71.1
RALF1-FLAG (processed)	9.5
FER^{ECD}-Citrine	75.7
