# Supplementary material for: Overlapping functions and protein-protein interactions of LRR-extensins in Arabidopsis
Source: PLoS Genet. 2020 Jun 19;16(6):e1008847. doi: 10.1371/journal.pgen.1008847 (PMC7357788; doi:10.1371/journal.pgen.1008847)
Supplement: S2 Table — (PDF) [file pgen.1008847.s010.pdf]

S2 Table Primers used for genotyping

Target Sequence	Primer	Sequence	
lrx1	LRX1_2800_F	GAAATCGATCCCGAGTCGTTG	*Bsi*EI digests the *lrx1* mutant
	LRX1_2980_R	TGGAGAGAAGATGTTGTAACATC	
lrx2	lrx2-1_F	TTGCGGATAATGATCTTGGAGGTTG	*Bcc*I digests the wild type
	lrx2-1_R	CTGCTTGGTATAACTCCTGTGAATC	
LRX3	LRX3_seq_F	TACTGTACCACACCGGTTTAAC	
	LRX3_LRR_R	AATTCTAGATCAACGGCCACAACTAAACGATC	
lrx3	LRX3_seq_F	TACTGTACCACACCGGTTTAAC	
	LBb1	GCGTGGACCGCTTGCTGCAACT	
LRX4	LRX4_seq_F	CCATAACCGGTTCCGGTTTGAG	
	LRX4_3'b	TTTAACAACAGAACGACCACAAC	
lrx4	LRX4_seq_F	CCATAACCGGTTCCGGTTTGAG	
	Gabi_Lb	GGGAATGGCGAAATCAAGGCATCG	
LRX5	LRX5_F2	GCTTGGTTTGTTAACAGATCTC	
	LRX5_3'	GCCGGAATCTTACCAGAGAATC	
lrx5	LRX5_F2	GCTTGGTTTGTTAACAGATCTC	
	LBb1	GCGTGGACCGCTTGCTGCAACT	
FER	FER_F1	GATTACTCTCCAACAGAGAAAATCCT	
	FER_R1	CGTATTGCTTTTCGATTTCCTA	
fer-4	FER_F2	ACGGTCTCAACGCTACCAAC	
	FER T-DNA	TTTCCCGCCTTCGGTTTA	
