# Supplementary material for: Overlapping functions and protein-protein interactions of LRR-extensins in Arabidopsis
Source: PLoS Genet. 2020 Jun 19;16(6):e1008847. doi: 10.1371/journal.pgen.1008847 (PMC7357788; doi:10.1371/journal.pgen.1008847)
Supplement: S3 Table — (PDF) [file pgen.1008847.s011.pdf]

S3 Table Primers used for cloning

Primer	Sequence
LRX1_XhoI_F	CTCGAGTTAGTAAAATTGGGTTTCTTGAC
LRX1_PstI_R	CTGCAGTCCACAGGGCGAGCAAG
LRX1_ΔLRR_SpeI_R	ACTAGTTCAGGGTACCGAATTCAAGTCCTC
LRX1_ΔNT_SalI_F	GTCGACCGAAAACCCGAGTCGTTGCTGGC
LRX1_ΔNT_SalI_R	CCGTCGACCATCGTCGTGGTCTGCTTTG
LRX1_Prom1000_F	AAAGTGAGGTATTTAGGTCATT
LRX3_KpnI_F	GACGGTACCTGTACACTGACATGAAGAAG
LRX3_PstI_R	CTGCAGTTTACCGGCGGACGAGACAAAAACG
LRX4oE_KpnI_F	GGTACCGTATCGTATCGTGAAATGAAGAAC
LRX4_PstI_R	CTGCAGTCCACCGAAGGCCGTG
LRX4_ΔLRR_PstI_R	GTTCTGCAGTTCCGGTTATCAAGAGCTTTAG
LRX4_ΔNT_F	CTAGGATCCAAAGCTCTTGATAACCGGAAG
LRX4_ΔNT_R	CTAGGATCCTGAGATTGAGAGAGAATGAGAG
Yeast two hybrid:	
LRX1_BamHI_F	GGATCCCTTCTCCTTCATACCCGAAAAC
LRX1_XhoI_R	CTCGAGTCAACTGCAATCCACAGGGCGAG
LRX2_BamHI_F	CAGGGATCCCTTCTCCTTCTAGTCCGAAAAC
LRX2_XhoI_R	GTCCTCGAGCTAACTACAATCAACAGAGCGTGAG
LRX3_BamHI_F	GGATCCTGGCGCTTGATAATCGGAAG
LRX3_ XhoI_R	ACGCTCGAGTCATCCACAATTTACCGGCGGAC
LRX5_BamHI_F	AGCGGATCCCGGCTTTAGATAACCGCCGAATCC
LRX5_XhoI_R	AGTCTCGAGTCAAAAGGAACCACAATCCACCG
RALF1_EcoRI_F	GAATTCAATAGAAGAATATTGGCGAC
RALF1_SmaI_R	CCCGGGTCAACTCCTGCAACGAATTTTGC
LxE1 constructs:	
LRX3_XhoI_F	CTCGAGATGAAGAAGACGATTCAAATC
LRX3_PstI_R	CTGCAGTTTACCGGCGGACGAGAC
LRX4_XhoI_F	GGTACCGTATCGTATCGTGAAATGAAGAAC
LRX4_PstI_R	CTGCAGTCCACCGAAGGCCGTG
LRX5_XhoI_F	CTCGAGATGAAGACGAAGATGATGATG
LRX5_PstI_R	TGACTGCAGCCACAATCCACCGGCGGAAGAG
LRX8_XhoI_F	TGACTCGAGATGACCCGAAGAACAATGGAG
LRX8_PstI_R	TGACTGCAGCTGCAATCCACAGGACGGC
LRX10_KpnI_F	AGAGGTACCGTTCCAAGTCATGACTAAACCTCC
LRX10_PstI_R	GATCTGCAGCTACAATCAACTGGACGGCTAATC
LRX11_XhoI_F	AGACTCGAGATGCCCTTCTATAAGCAGCC
LRX11_PstI_R	TCTCTGCAGCTACAATCAACCGGGCGGTTGCTA
