# Supplementary material for: Overlapping functions and protein-protein interactions of LRR-extensins in Arabidopsis
Source: PLoS Genet. 2020 Jun 19;16(6):e1008847. doi: 10.1371/journal.pgen.1008847 (PMC7357788; doi:10.1371/journal.pgen.1008847)
Supplement: S4 Table — (PDF) [file pgen.1008847.s012.pdf]

S4 Table Primers used for RT-PCR

Target Sequence	Primer	Sequence
L1E1	LRX1 cmyc F	GAAATCGATCCCGAGTCGTTG
	LRX1_Ext_R	TGGAGAGAAGATGTTGTAACATC
L3E1	LRX3_seq_F	TACTGTACCACACCGGTTTAAC
	LRX1_Ext_R	TGGAGAGAAGATGTTGTAACATC
L4E1	LRX4_seq_F	CCATAACCGGTTCCGGTTTGAG
	LRX1_Ext_R	TGGAGAGAAGATGTTGTAACATC
L5E1	LRX5_F2	GCTTGGTTTGTTAACAGATCTC
	LRX1_Ext_R	TGGAGAGAAGATGTTGTAACATC
